# Supplementary material for: ASCOT identifies key regulators of neuronal subtype-specific splicing
Source: Nat Commun. 2020 Jan 9;11:137. doi: 10.1038/s41467-019-14020-5 (PMC6952364; doi:10.1038/s41467-019-14020-5)
Supplement: Supplementary file 2 — Description of Additional Supplementary Files [file 41467_2019_14020_MOESM2_ESM.pdf]

Description of Additional Supplementary Files

File Name: Supplementary Data 1
Description: PSI table of neuronal cell type-specific alternative exons Examples of neuronal cell type-specific exons identified in mouse RNA-Seq datasets (MESA)

File Name: Supplementary Data 2
Description: PSI table of tissue-specific alternative exons Examples of tissue -specific exons identified in human RNA-Seq datasets (GTEx)

File Name: Supplementary Data 3
Description: PSI table of exons activated by overexpression of *MSI1* Table of alternative exons (enriched in rods and neurons) and cryptic exons that are upregulated due to overexpression of *MSI1*.
