# Supplementary material for: ASCOT identifies key regulators of neuronal subtype-specific splicing
Source: Nat Commun. 2020 Jan 9;11:137. doi: 10.1038/s41467-019-14020-5 (PMC6952364; doi:10.1038/s41467-019-14020-5)
Supplement: Supplementary file 7 — Supplementary Information [file 41467_2019_14020_MOESM7_ESM.pdf]

Supplementary Information

ASCOT identifies key regulators of neuronal subtype-specific splicing

Ling et al.

Supplemental File Table of Contents

Associated with Figure 1.

Supplemental Figure 1. Diagram of “junction-walking” strategy for alternative splicing analysis

Supplemental Figure 2. Binary decision splicing events represent most local splicing variation

Supplemental Figure 3. Rods do not express many of the common neuronal splicing factors

Supplemental Figure 4. RTPCR validation of novel alternative exons identified with ASCOT

Associated with Figure 2.

Supplemental Figure 5. Rod-specific exons, both previously identified and novel

Supplemental Figure 6. Alternative splicing analysis of single-cell RNA-Seq data derived from full-length transcripts

Associated with Figure 4.

Supplemental Figure 7. Activation of rod-specific exons in HepG2 cells requires FACS isolation of the most strongly expressing cells

Supplemental Figure 8. Increased *MSI1* expression and *PTBP1* downregulation may interact synergistically to activate rod/neuronal alternative exons

Supplemental Figure 9. Upstream and downstream flanking sequences for rod-specific exons

Supplemental Figure 10. *Msi1* CLIP-Seq supports direct interaction with photoreceptor-specific exons

Associated with Figure 5.

Supplemental Figure 11. *Msi1* is necessary for rod photoreceptor-specific splicing

Associated with Discussion

Supplemental Figure 12. Many of the exons detected by ASCOT are unannotated

Supplemental Figure 13. Dataset clustering can improve the detection of binary splicing events

Supplemental Figure 14. Rod-specific exons in the SRAv2 Snaptron compilation

Supplementary References

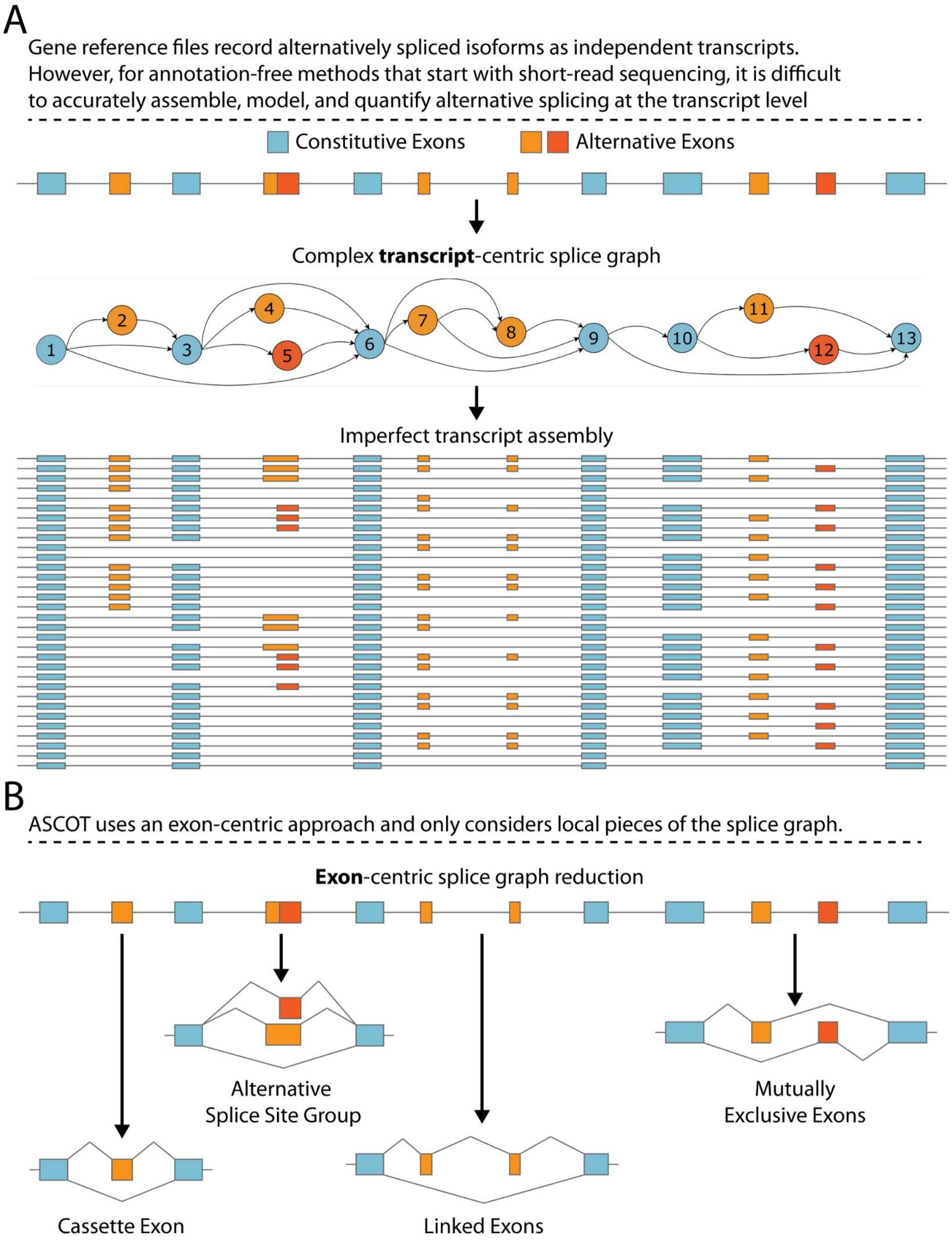
A
Gene reference files record alternatively spliced isoforms as independent transcripts.
However, for annotation-free methods that start with short-read sequencing, it is difficult
to accurately assemble, model, and quantify alternative splicing at the transcript level
Constitutive Exons
Alternative Exons
Complex **transcript**-centric splice graph
1
2
3
4
5
6
7
8
9
10
11
12
13
Imperfect transcript assembly
B
ASCOT uses an exon-centric approach and only considers local pieces of the splice graph.
Exon-centric splice graph reduction
Alternative
Splice Site Group
Mutually
Exclusive Exons
Cassette Exon
Linked Exons

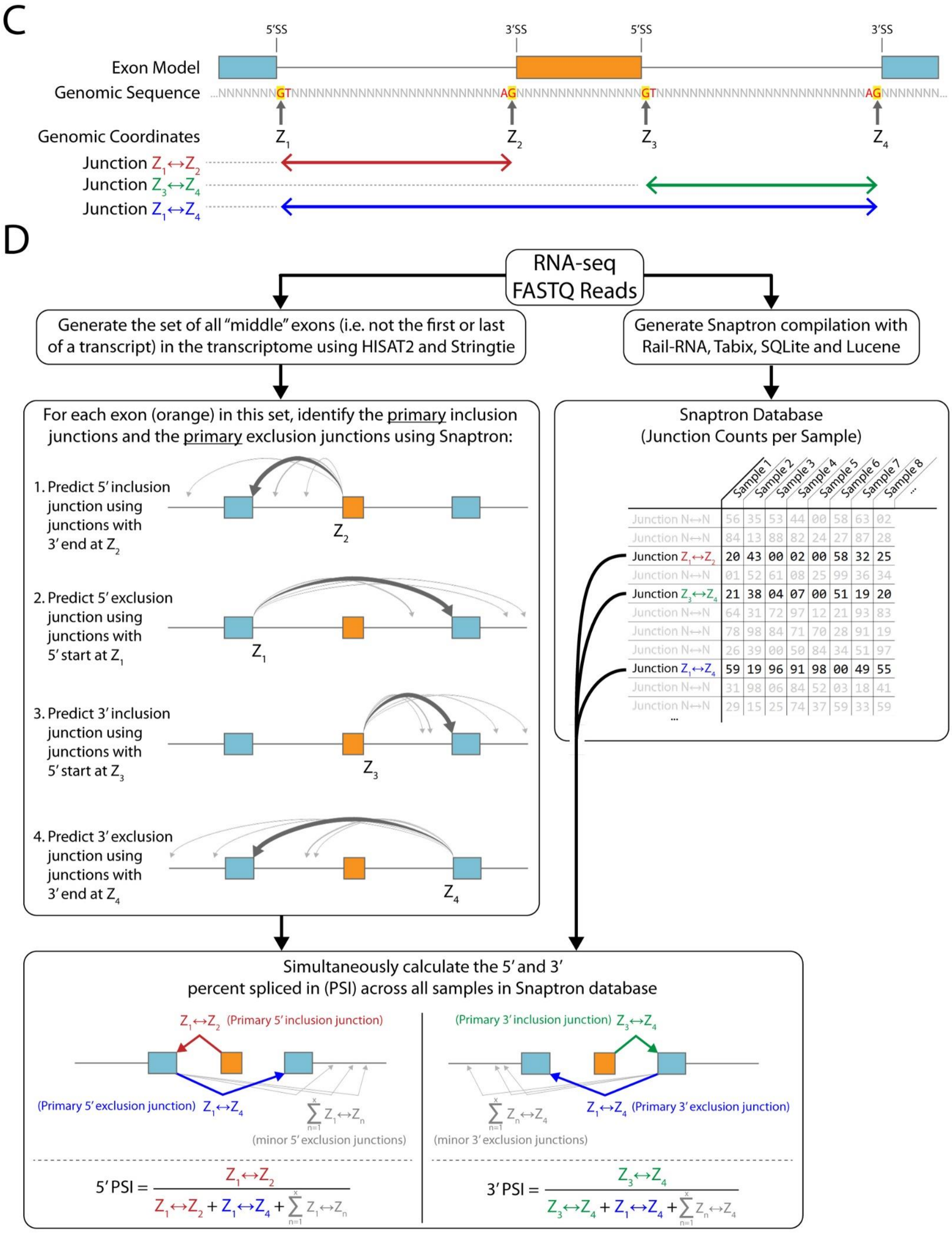
C
5'SS
3'SS
5'SS
3'SS
Exon Model
Genomic Sequence
Genomic Coordinates
Z1
Z2
Z3
Z4
Junction Z1↔Z2
Junction Z3↔Z4
Junction Z1↔Z4
D
RNA-seq FASTQ Reads
Generate the set of all "middle" exons (i.e. not the first or last of a transcript) in the transcriptome using HISAT2 and Stringtie
Generate Snaptron compilation with Rail-RNA, Tabix, SQLite and Lucene
For each exon (orange) in this set, identify the primary inclusion junctions and the primary exclusion junctions using Snaptron:
1. Predict 5' inclusion junction using junctions with 3' end at Z2
2. Predict 5' exclusion junction using junctions with 5' start at Z1
3. Predict 3' inclusion junction using junctions with 5' start at Z3
4. Predict 3' exclusion junction using junctions with 3' end at Z4
Snaptron Database
(Junction Counts per Sample)
Sample 1 Sample 2 Sample 3 Sample 4 Sample 5 Sample 6 Sample 7 Sample 8 ...
Junction N↔N 56 35 53 44 00 58 63 02
Junction N↔N 84 13 88 82 24 27 87 28
Junction Z1↔Z2 20 43 00 02 00 58 32 25
Junction N↔N 01 52 61 08 25 99 36 34
Junction Z3↔Z4 21 38 04 07 00 51 19 20
Junction N↔N 64 31 72 97 12 21 93 83
Junction N↔N 78 98 84 71 70 28 91 19
Junction N↔N 26 39 00 50 84 34 51 97
Junction Z1↔Z4 59 19 96 91 98 00 49 55
Junction N↔N 31 98 06 84 52 03 18 41
Junction N↔N 29 15 25 74 37 59 33 59
...
Simultaneously calculate the 5' and 3' percent spliced in (PSI) across all samples in Snaptron database
Z1↔Z2 (Primary 5' inclusion junction)
(Primary 5' exclusion junction) Z1↔Z4
Σ Z1↔Zn (minor 5' exclusion junctions)
(Primary 3' inclusion junction) Z3↔Z4
Z1↔Z4 (Primary 3' exclusion junction)
Σ Zn↔Z4 (minor 3' exclusion junctions)
5' PSI = Z1↔Z2 / (Z1↔Z2 + Z1↔Z4 + Σ Z1↔Zn)
3' PSI = Z3↔Z4 / (Z3↔Z4 + Z1↔Z4 + Σ Zn↔Z4)

E

Classification Conditions

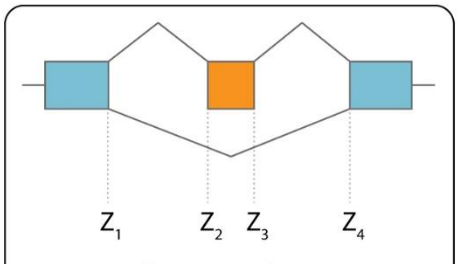

Cassette Exon

A cassette is defined as an exon that has equivalent 5′ and 3′ exclusion junctions (Z_1-Z_4)

Junction walking:
$Z_2 \rightarrow Z_1 \rightarrow Z_4$ and $Z_3 \rightarrow Z_4 \rightarrow Z_1$

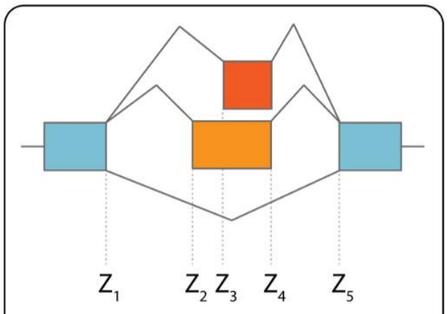

Alternative Splice Site Group

A group of exons define an alternative splite site exon group if they share the same 3′ inclusion junction (Z_4-Z_5) and have equivalent 5′ and 3′ exclusion junctions (Z_1-Z_5)

Junction walking:
$Z_3 \rightarrow Z_1 \rightarrow Z_5$ and $Z_4 \rightarrow Z_5 \rightarrow Z_1$
$Z_2 \rightarrow Z_1 \rightarrow Z_5$ and $Z_4 \rightarrow Z_5 \rightarrow Z_1$

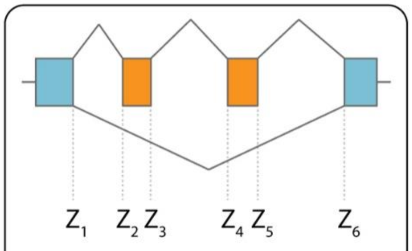

Linked Exons

Exons are linked if they share equivalent 5′ and 3′ exclusion junctions (Z_1-Z_6) but have inclusion junctions that end between Z_3 and Z_4

Junction walking:
$Z_2 \rightarrow Z_1 \rightarrow Z_6$ and $Z_5 \rightarrow Z_6 \rightarrow Z_1$
$Z_3 \rightarrow Z_4$ and $Z_4 \rightarrow Z_3$, otherwise complex splicing between Z_2 and Z_5

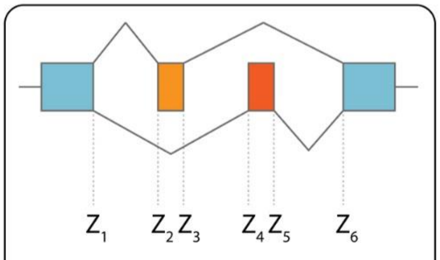

Mutually Exclusive Exons

Exons are mutually exclusive if they have high confidence inclusion junctions that extend to the same boundary splice sites (Z_1 and Z_6)

Junction walking:
$Z_2 \rightarrow Z_1$ and $Z_4 \rightarrow Z_1$
$Z_3 \rightarrow Z_6$ and $Z_5 \rightarrow Z_6$

Supplemental Figure 1. Diagram of "junction-walking" strategy for identifying alternative splicing patterns using a splice junction count table.

(A) Alternative splicing introduces a large amount transcriptomic and proteomic diversity. Most exons can be generally classified as either constitutive (spliced into mRNA for nearly all cellular environments) or alternative (spliced in only under certain conditions). Constitutive exons are labeled blue and alternative exons are labeled as shades of orange. Complex splice graphs can be derived from split-reads and a variety of mRNA transcripts are generated from these graphs. When analyzing alternative splicing with a gene reference file, spliced isoforms are recorded and quantified as independent transcripts. However, it is difficult for annotation-free methods to accurately assemble, model, and quantify alternative splicing with short-read sequencing. This is especially true for transcripts with distant alternative exons. Low abundance, spurious isoforms can also be reported as alternatively spliced and it is often difficult for biologists to interpret which transcripts are biologically relevant.

(B) ASCOT uses an exon-centric approach to reduce this complexity by only considering local regions of the splice graph and analyzing these elements independently from one another. In this work, we focus on four binary splicing decisions: cassette exons, alternative splice site exon groups that share the same exclusion junction, linked exons, and mutually exclusive pairs of exons.

(C) Our method for splicing analysis relies on evidence from RNA-Seq split-read alignments (i.e. splice junctions), as opposed to coverage. Split-reads are generally very reliable measurements, since sequences that contain two different regions of the genome are likely to have been joined via the spliceosome. Split-read alignment accuracy also benefits from requiring known splice motifs at either end of an intron. We can therefore easily identify which sets of junctions share the same start or end coordinates. In this diagram, inclusion junctions are shown in red ($Z_1 \leftrightarrow Z_2$) and green ($Z_3 \leftrightarrow Z_4$) while the exclusion junction is shown in blue ($Z_1 \leftrightarrow Z_4$). For these inclusion junctions, Z_2 and Z_3 are adjacent to the alternative exon (orange), but no information links Z_1 and Z_4 unless we know that $Z_1 \leftrightarrow Z_4$ is the exclusion junction. This forms a closed loop since we can start from any coordinate and trace a path through an alternating series of exons and introns that leads to the starting coordinate, e.g. start at Z_1, follow the 5' inclusion junction to get to Z_2, follow the alternative exon to get to Z_3, follow the 3' inclusion junction to get to Z_4, then follow the exclusion junction to get back to Z_1. If identical loops are derived from the 5' and 3' inclusion junctions, there is high confidence that the alternative exon is a cassette exon. We developed a "junction-walking" strategy to identify these closed junction loops both across the transcriptome and across tens of thousands

of summarized RNA-Seq samples by using the coordinates and count tables of a Snaptron[1] splice junction database. Because we use Snaptron queries in this process, the strategy is informed by junction evidence in all samples, and not just a single simple.

(D) The flowchart of our method begins with a set of raw RNA-Seq fastq reads. First, all the raw reads from all the input accessions were analyzed using the Rail-RNA spliced alignment program. Rail-RNA outputs a few summaries, the relevant here being a table of splice-junction evidence. In this table, each row is a splice junction and each column is an individual run accession. The elements of the table give the number of times a spliced alignment from an individual (column) spanned a junction (row). This summary was then indexed using Tabix and SQLite, and all the associated metadata for the run accessions was indexed using Lucene, to form a complete Snaptron compilation, a queryable database. In parallel with this process, we also analyzed every sample of raw RNA-SEQ Fastq reads with StringTie and identified the set of all "middle" exons by excluding all first and last exons. In theory, this set of middle exons contains the coordinates for all possible internal alternative exons that would be suitable queries for our junction-walking strategy. Next, for each middle exon, we predict the 5' and 3' inclusion junctions by querying Snaptron for all junctions ending at Z_2 (for the 5' inclusion junction) or beginning at Z_3 (for the 3' inclusion junction). We must also establish that if this is an alternative exon, there is only one primary 5' or 3' inclusion junction. For the 5' inclusion junction, we set a threshold where the primary inclusion junction must represent >70% of all junction counts (across all samples) that end at Z_2; a similar threshold is set for the 3' inclusion junction and junctions that begin at Z_3. We then calculate the primary 5' and 3' exclusion junctions by identifying all junctions that begin at Z_1 (for the 5' exclusion junction) and all junctions that end at Z_4 (for the 3' exclusion junction). We then test whether a junction loop has been closed using logic outlined in section (**E**). For exons that have consistent 5' and 3' closed junction loops, we can then easily calculate 5' and 3' percent spliced-in (PSI) values for each alternative exon with Snaptron per the formulas shown in the figure. For example, for the 5'PSI, $Z_1 \leftrightarrow Z_2$ represents a vector of 5' inclusion junction counts for each sample in the Snaptron database. Similarly, $Z_1 \leftrightarrow Z_4$ represents an equivalent count vector for the exclusion junction, and the sum of $Z_1 \leftrightarrow Z_n$ represents the sum of vectors for every other possible exclusion junction that begins at Z_1. This sum of minor exclusion junctions is important to ensure an accurate PSI calculation. Equivalent calculations are also performed to find the 3' PSI for each sample. Finally, the PSI value for each exon is reported as the average of the 5' PSI and 3' PSI values. Since differences between 5' and 3' PSI can be attributed to various technical confounders such as GC bias & random hexamer priming, we used a conservative

threshold where the difference between 5’ and 3’PSI is < 0.8 (using a PSI scale from 0 to 1). We believe that this threshold excludes a reasonably large fraction of false positives, while maintaining the comprehensiveness of ASCOT. Furthermore, investigators who wish to adjust this threshold for higher or lower stringency can do so using the --f parameter in ascot_psi.py, as documented in the github repository.

(E) In this work, we identify four closed junction loops that are essentially decisions between two possible splicing outcomes (binary decisions): 1. Cassette Exons, 2. Alternative Splice Site Exon Groups, 3. Linked Exons, and 4. Mutually Exclusive Exons. In the simplest case, cassette exons are identified when the primary 5’ and 3’ exclusion junctions are identical. However, in some cases multiple exons can share the exclusion junction but possess alternative 5’ or 3’ splice sites. By treating these exons as a group, we can again model the splicing as a binary decision: either using any of the exons or using none. Further analysis can determine which specific splice sites are used within the group, but for the current work we focus on the primary splicing decision because measuring alternative splice site usage within this exon group would be treated as a nested decision. We also identify linked exons by finding an exon pair where the 5’ exon’s 5’ exclusion junction is identical the 3’ exon’s 3’ exclusion junction. It is important to note that our junction walking strategy is unable to determine what lies in between the exons. Given the possibility that nested splicing decisions exists between Z_3 and Z_4, we do not currently analyze splicing between linked exons. Finally, we also identify mutually exclusive exons by finding exon pairs where both exons inclusion junctions terminate at the same coordinates, but the 5’ exon’s inclusion junctions are the 3’ exon’s exclusion junctions and vice versa.

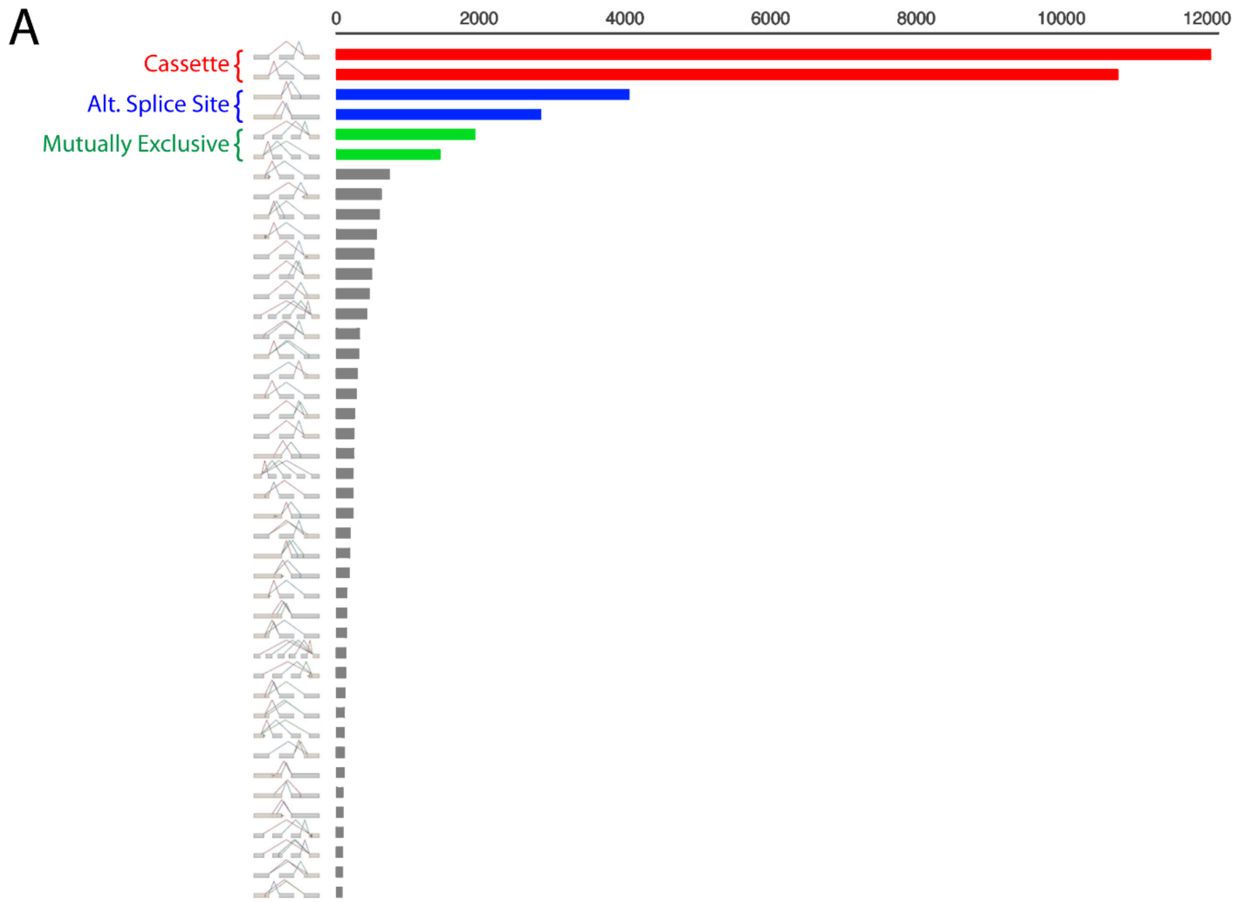

Supplemental Figure 2. Binary decision splicing events represent most local splicing variation. (**A**) Exonic splicing variation across the genome as detected by MAJIQ, adapted from Figure 4 of Vaquero-Garcia et al.[2] Simple alternative splicing decisions such as cassette exons, alternative splice sites, and mutual exclusive exons represent most local splicing variation.

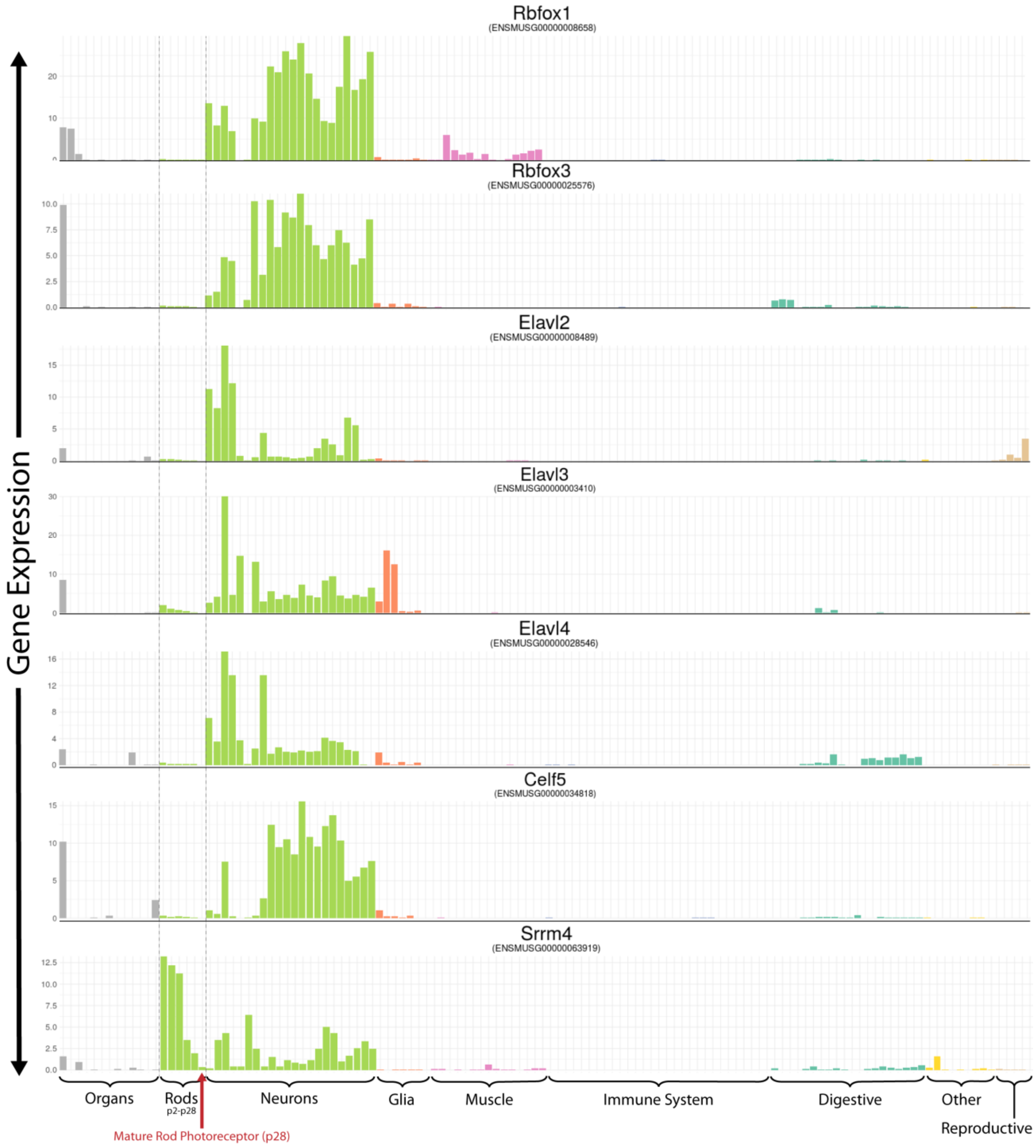

Supplemental Figure 3. Rods do not express many of the common neuronal splicing factors. *Rbfox1*, *Rbfox3*, *Elavl2*, *Elavl3*, *Elavl4*, *Celf5*, and *Srrm4* are splicing factors that are selectively expressed in most neurons. Rod photoreceptors, however, do not express these genes.

A

Gene	Exon coordinates (mm10)	Cell type specificity predicted by ASCOT
Kctd5	chr17:24053272-24053330	Photoreceptors
Flna	chrX:74240816-74240872	All neurons
Flnb	chr14:7886026-7886123	All neurons
Itgb1	chr8:128726983-128727063	All muscles
Ywhae	chr11:75762698-75762742	Skeletal muscle
Vcl	chr14:21027083-21027286	Skeletal muscle and colon

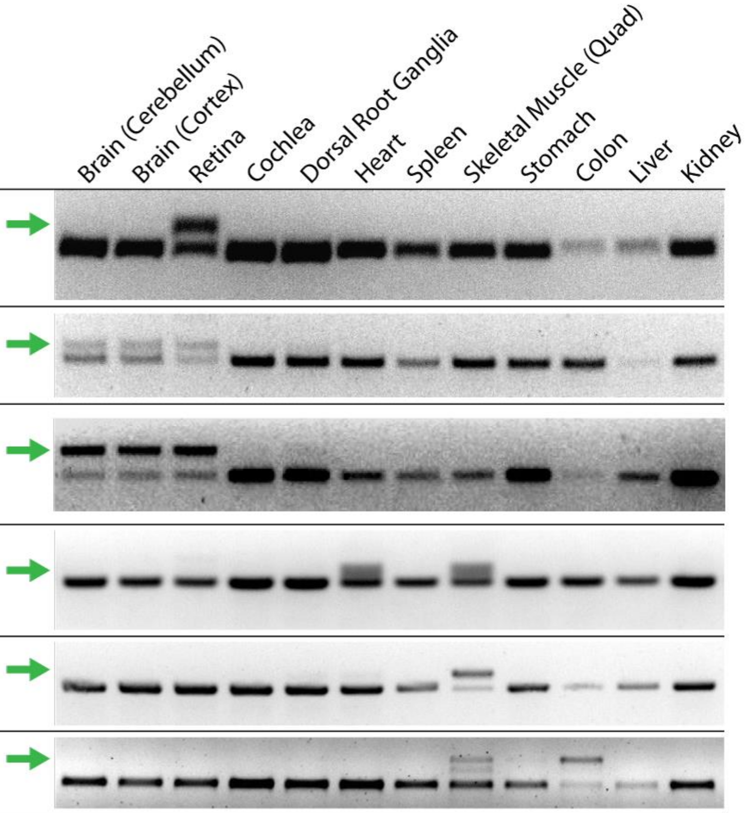

B

Gene	Exon coordinates (mm10)	Cell type specificity predicted by ASCOT
Farp2	chr1:93617298-93617318	Photoreceptors
Cltc	chr11:86713425-86713436	Photoreceptors
Ptprf	chr4:118221571-118221582	DRG and photoreceptors
Ank3	chr10:69823078-69823101	All neurons
Ppp6r3	chr19:3459747-3459764	All neurons

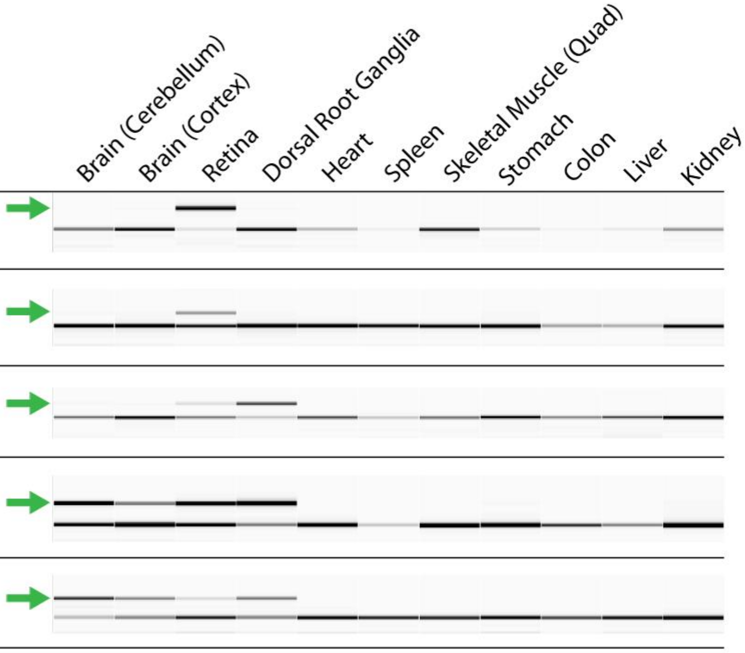

Supplemental Figure 4. RT-PCR validation of novel, unannotated exons identified with ASCOT. Using PSI data available through ASCOT, we identified unannotated (as of GENCODE release M20) cell type-specific exons that reside in ubiquitously expressed genes. We then designed primers spanning these alternative exons to yield a constitutive RT-PCR product and a larger RT-PCR product that contains the spliced-in cell specific exon (green arrows). RT-PCR across a panel of tissues confirms the cell type-specificity predicted by ASCOT. (**A**) We used 2% agarose gels to resolve large size differences between RT-PCR products. (**B**) For smaller differences between RT-PCR products (<30bp), we used an Agilent Fragment Analyzer.

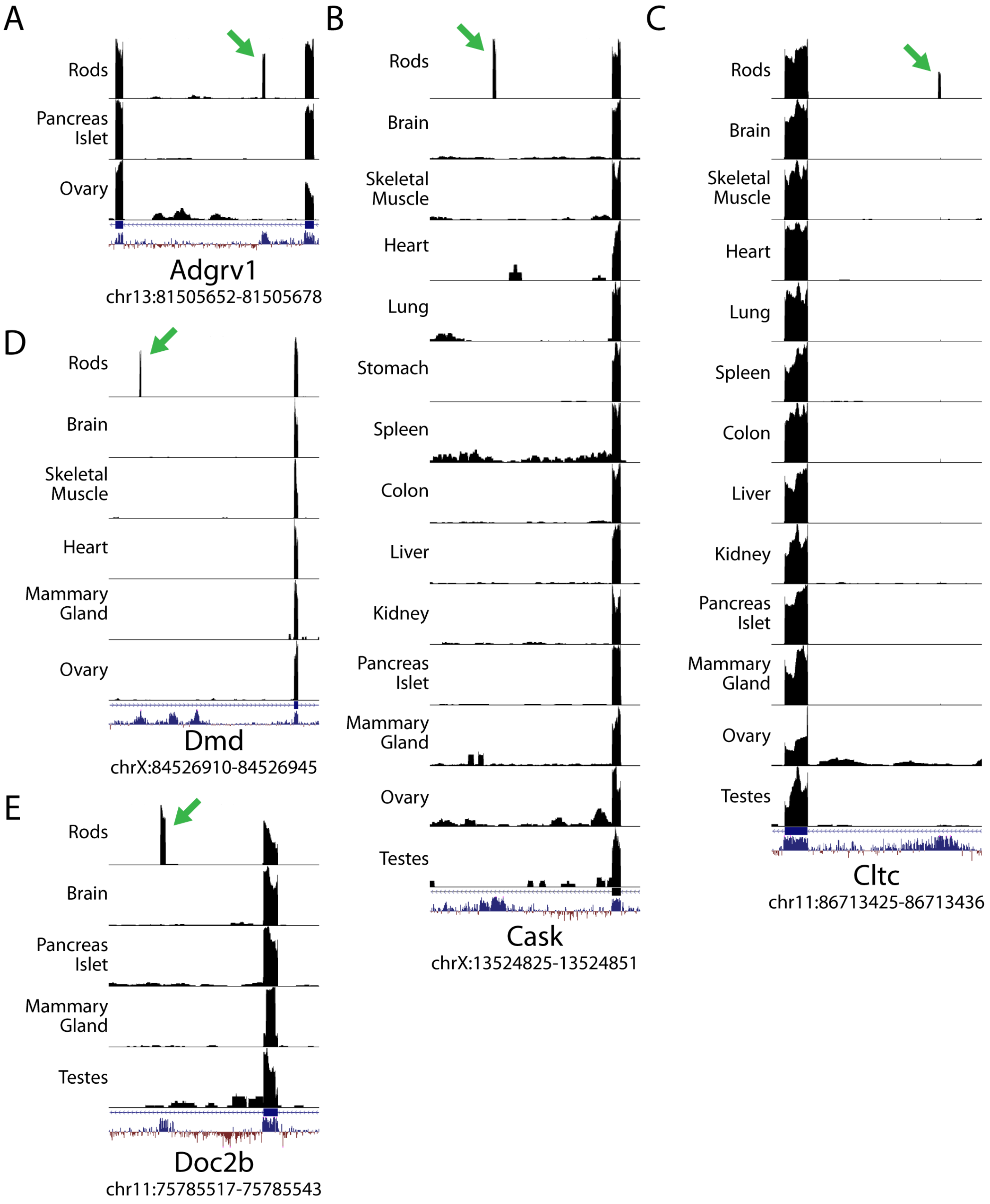
A
Rods
Pancreas Islet
Ovary
Adgrv1
chr13:81505652-81505678
B
Rods
Brain
Skeletal Muscle
Heart
Lung
Stomach
Spleen
Colon
Liver
Kidney
Pancreas Islet
Mammary Gland
Ovary
Testes
Cask
chrX:13524825-13524851
C
Rods
Brain
Skeletal Muscle
Heart
Lung
Spleen
Colon
Liver
Kidney
Pancreas Islet
Mammary Gland
Ovary
Testes
Cltc
chr11:86713425-86713436
D
Rods
Brain
Skeletal Muscle
Heart
Mammary Gland
Ovary
Dmd
chrX:84526910-84526945
E
Rods
Brain
Pancreas Islet
Mammary Gland
Testes
Doc2b
chr11:75785517-75785543

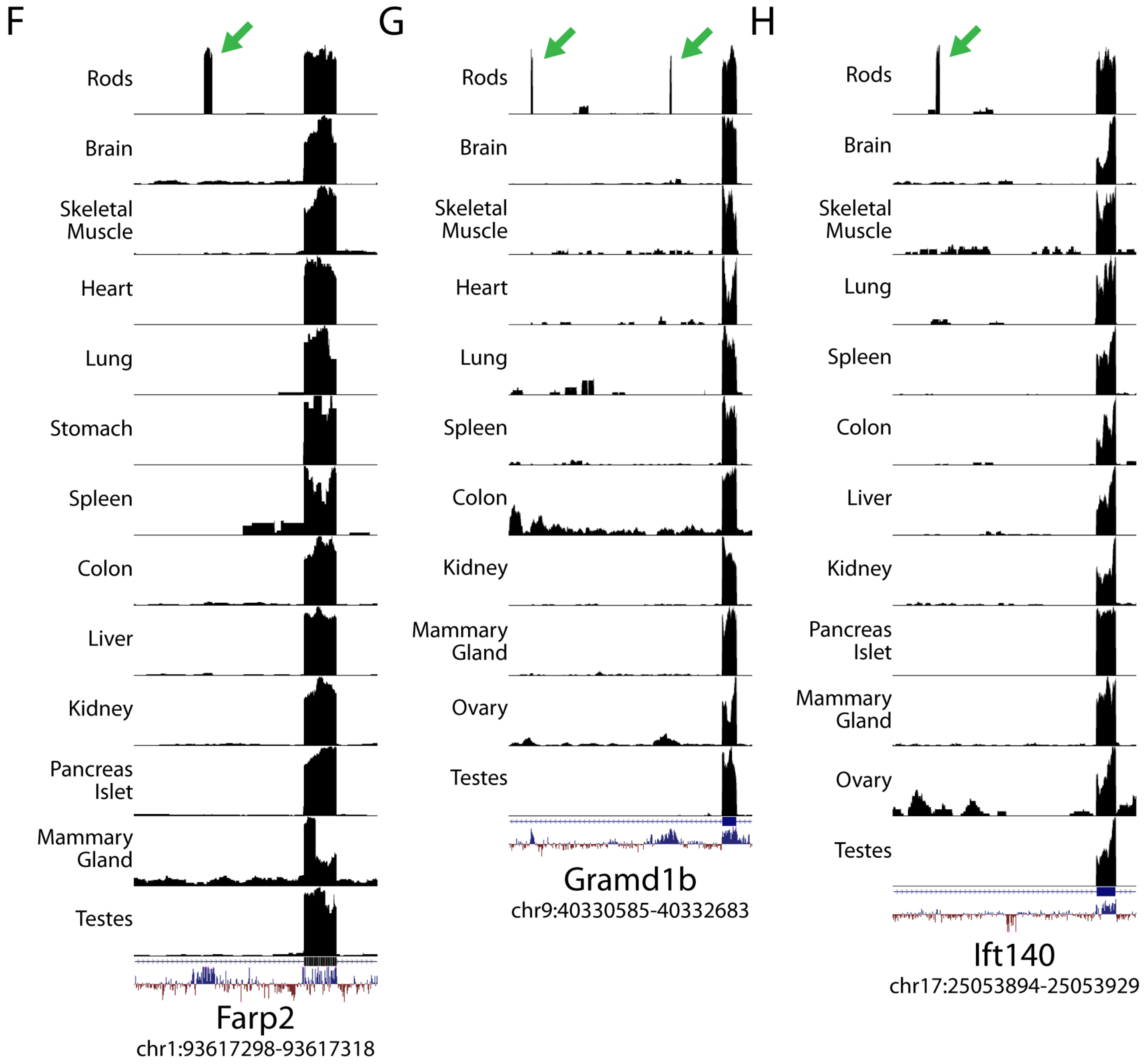
F
Rods
Brain
Skeletal Muscle
Heart
Lung
Stomach
Spleen
Colon
Liver
Kidney
Pancreas Islet
Mammary Gland
Testes
Farp2
chr1:93617298-93617318
G
Rods
Brain
Skeletal Muscle
Heart
Lung
Spleen
Colon
Kidney
Mammary Gland
Ovary
Testes
Gramd1b
chr9:40330585-40332683
H
Rods
Brain
Skeletal Muscle
Lung
Spleen
Colon
Liver
Kidney
Pancreas Islet
Mammary Gland
Ovary
Testes
Ift140
chr17:25053894-25053929

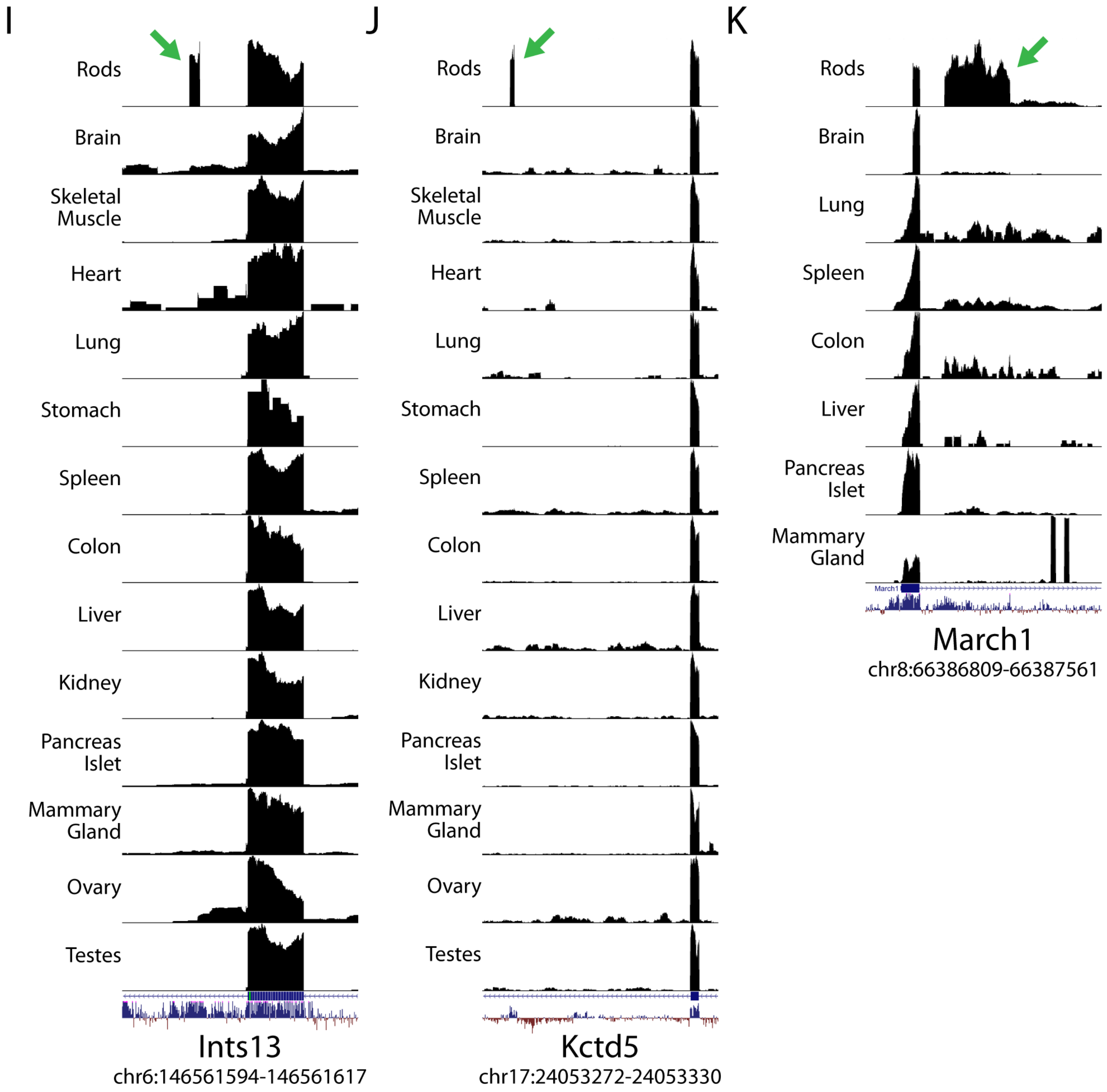
I
Rods
Brain
Skeletal Muscle
Heart
Lung
Stomach
Spleen
Colon
Liver
Kidney
Pancreas Islet
Mammary Gland
Ovary
Testes
Ints13
chr6:146561594-146561617
J
Rods
Brain
Skeletal Muscle
Heart
Lung
Stomach
Spleen
Colon
Liver
Kidney
Pancreas Islet
Mammary Gland
Ovary
Testes
Kctd5
chr17:24053272-24053330
K
Rods
Brain
Lung
Spleen
Colon
Liver
Pancreas Islet
Mammary Gland
March1
chr8:66386809-66387561

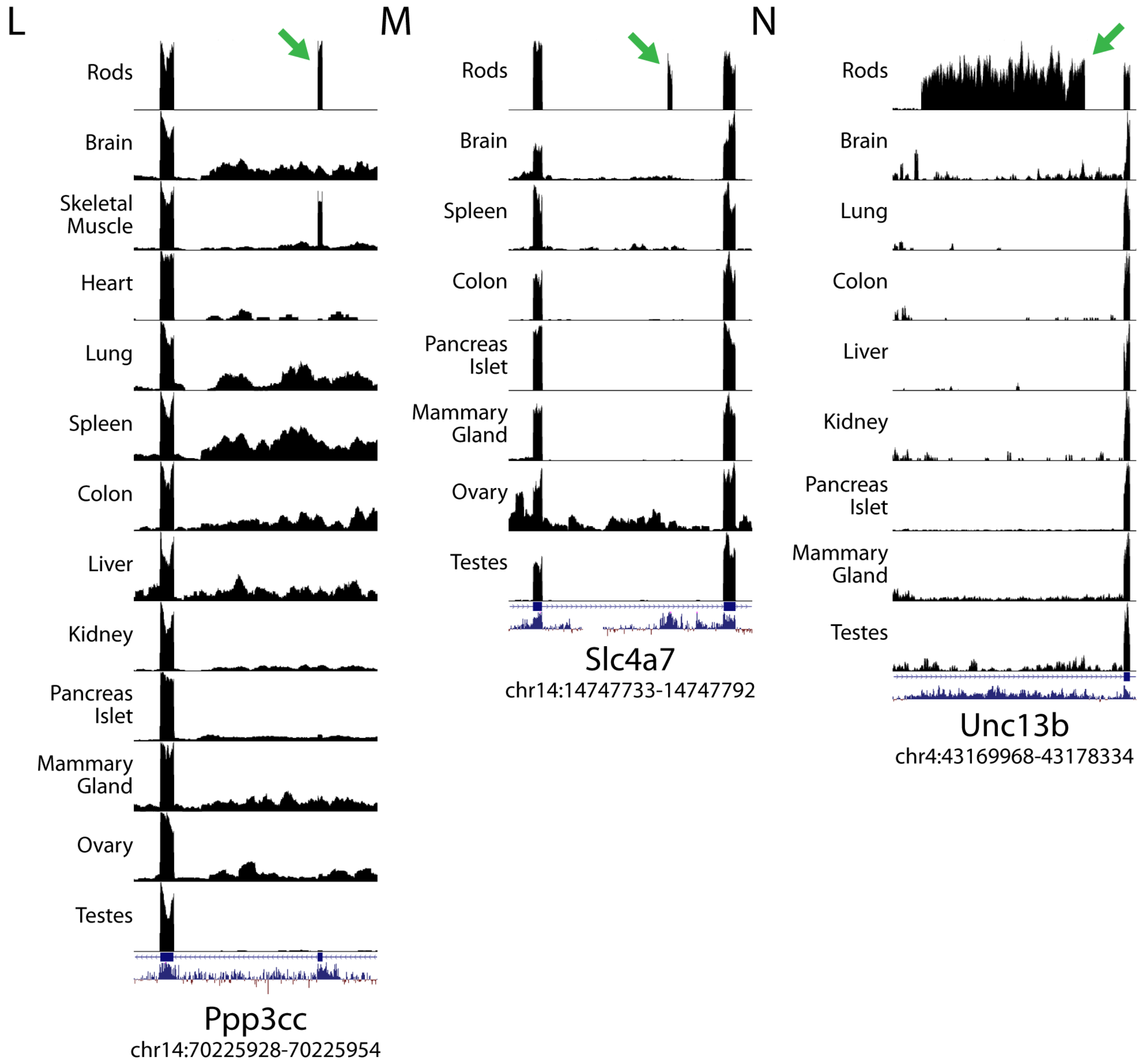
L
Rods
Brain
Skeletal Muscle
Heart
Lung
Spleen
Colon
Liver
Kidney
Pancreas Islet
Mammary Gland
Ovary
Testes
Ppp3cc
chr14:70225928-70225954
M
Rods
Brain
Spleen
Colon
Pancreas Islet
Mammary Gland
Ovary
Testes
Slc4a7
chr14:14747733-14747792
N
Rods
Brain
Lung
Colon
Liver
Kidney
Pancreas Islet
Mammary Gland
Testes
Unc13b
chr4:43169968-43178334

Supplemental Figure 5. Rod-specific exons, both previously identified and novel

Of the 31 rod-specific alternative splicing events that are shared between mouse and human, 17 have been previously reported (*Amph*[3,4], *Atp1b2*[4,5], *Plekhb1*[4,6], *Bsg*[4,7–9], *Ttc8*[4,10,11], *Impdh1*[4,12], *Cc2d2a*[4], *Kif1b*[4], *Efr3a*[4], *Hcn1*[4], *Unc13b*[4], *Kmt2d*[4], *Man2a2*[4], *Msi2*[4], *Stxbp5*[4]) while 14 novel rod-specific splicing events have not been previously reported (*Adgrv1*, *Cask*, *Cltc*, *Dmd*, *Doc2b*, *Farp2*, *Gramd1b*, *Ift140*, *Ints13*, *Kctd5*, *March1*, *Ppp3cc*, *Slc4a7*, and a second exon in *Unc13b*). Shown in this figure are UCSC bigwig visualizations of the novel rod exons comparing rods to other mouse tissues. Tissues in which the gene is not expressed are excluded from the visualization.

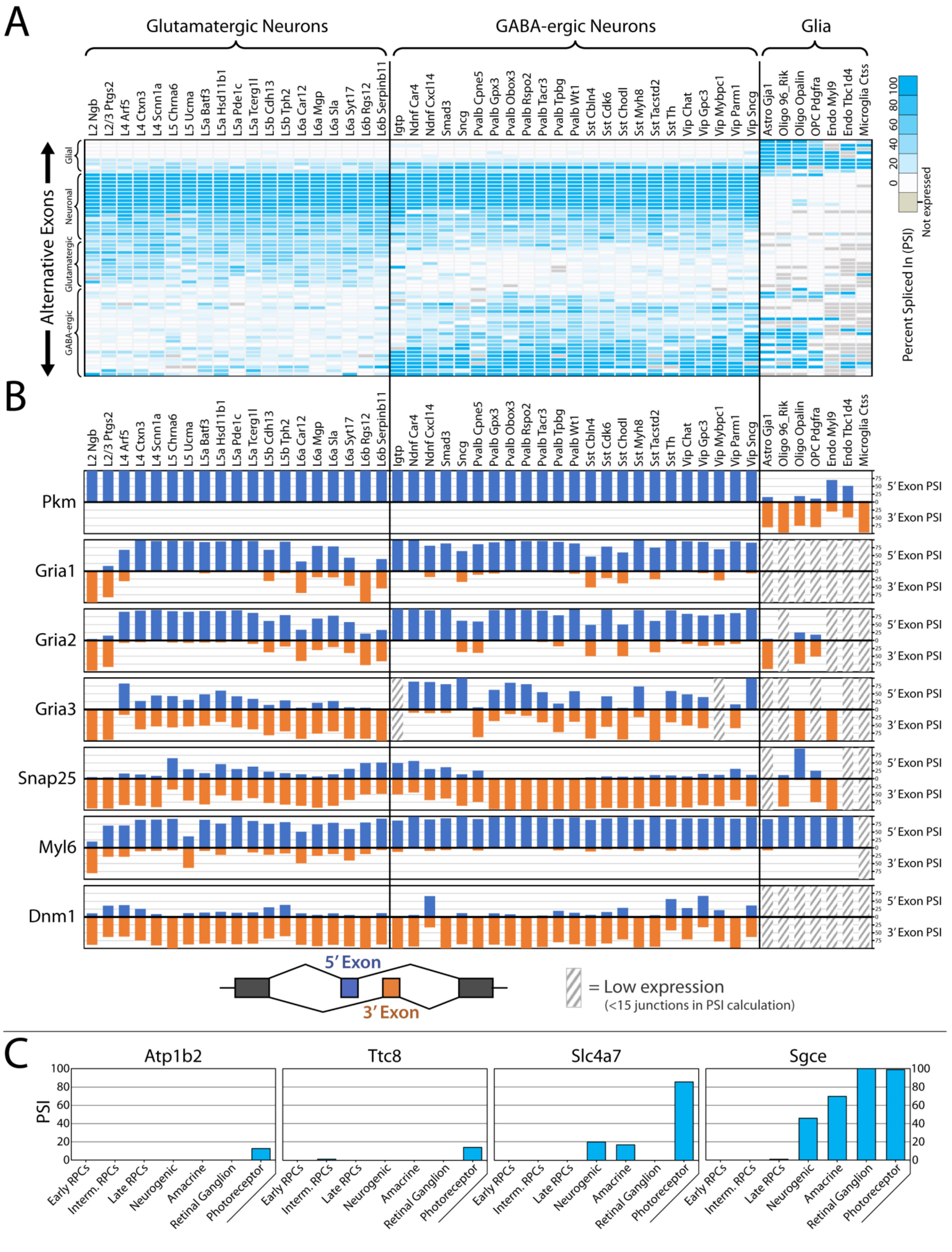

A
Glutamatergic Neurons
GABA-ergic Neurons
Glia
L2 Ngb
L2/3 Ptgs2
L4 Arf5
L4 Ctxn3
L4 Scnn1a
L5 Chrna6
L5 Ucma
L5a Batf3
L5a Hsd11b1
L5a Pde1c
L5a Tcerg1l
L5b Cdh13
L5b Tph2
L6a Car12
L6a Mgp
L6a Sla
L6a Syt17
L6b Rgs12
L6b Serpinb11
Igtp
Ndnf Car4
Ndnf Cxcl14
Smad3
Sncg
Pvalb Cpne5
Pvalb Gpx3
Pvalb Obox3
Pvalb Rspo2
Pvalb Tacr3
Pvalb Tpbg
Pvalb Wt1
Sst Cbln4
Sst Cdk6
Sst Chodl
Sst Myh8
Sst Tacstd2
Sst Th
Vip Chat
Vip Gpc3
Vip Mybpc1
Vip Parm1
Vip Sncg
Astro Gja1
Oligo 96_Rik
Oligo Opalin
OPC Pdgfra
Endo Myl9
Endo Tbc1d4
Microglia Ctss
Alternative Exons
Glial
Neuronal
Glutamatergic
GABA-ergic
100 80 60 40 20 0
Not expressed
Percent Spliced In (PSI)
B
Pkm
Gria1
Gria2
Gria3
Snap25
Myl6
Dnm1
5′ Exon PSI
3′ Exon PSI
5′ Exon
3′ Exon
= Low expression
(<15 junctions in PSI calculation)
C
Atp1b2
Ttc8
Slc4a7
Sgce
PSI
100 80 60 40 20 0
Early RPCs
Interm. RPCs
Late RPCs
Neurogenic
Amacrine
Retinal Ganglion
Photoreceptor

D

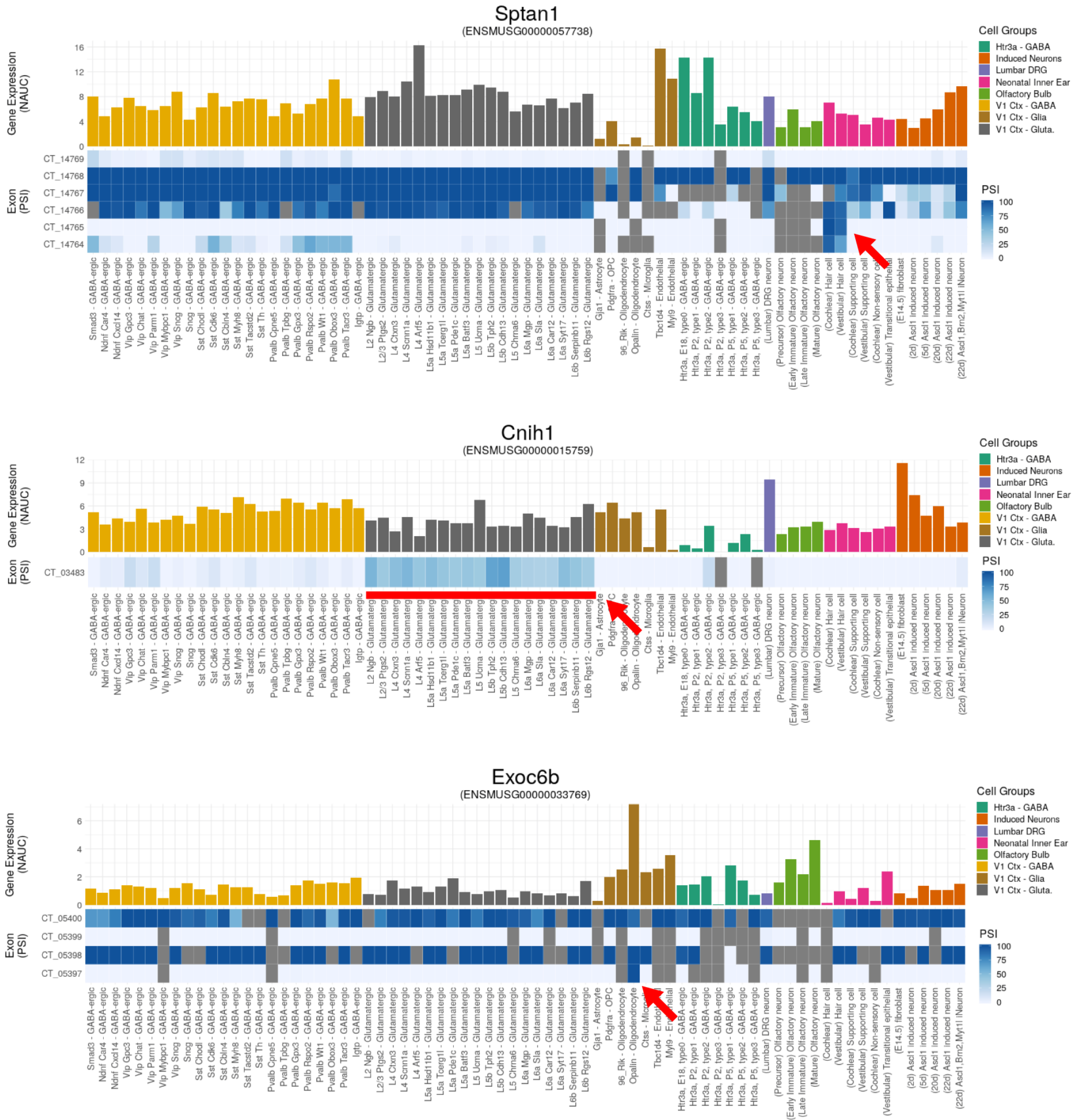

Sptan1
(ENSMUSG00000057738)
Gene Expression (NAUC)
Exon (PSI)
CT_14769
CT_14768
CT_14767
CT_14766
CT_14765
CT_14764
Cell Groups
Htr3a - GABA
Induced Neurons
Lumbar DRG
Neonatal Inner Ear
Olfactory Bulb
V1 Ctx - GABA
V1 Ctx - Glia
V1 Ctx - Gluta.
PSI
100
75
50
25
0
Smad3 - GABA-ergic
Ndnf Car4 - GABA-ergic
Ndnf Cxcl14 - GABA-ergic
Vip Gpc3 - GABA-ergic
Vip Chat - GABA-ergic
Vip Parm1 - GABA-ergic
Vip Mybpc1 - GABA-ergic
Vip Sncg - GABA-ergic
Sncg - GABA-ergic
Sst Chodl - GABA-ergic
Sst Cdk6 - GABA-ergic
Sst Cbln4 - GABA-ergic
Sst Myh8 - GABA-ergic
Sst Tacstd2 - GABA-ergic
Sst Th - GABA-ergic
Pvalb Cpne5 - GABA-ergic
Pvalb Tpbg - GABA-ergic
Pvalb Gpx3 - GABA-ergic
Pvalb Rspo2 - GABA-ergic
Pvalb Wt1 - GABA-ergic
Pvalb Obox3 - GABA-ergic
Pvalb Tacr3 - GABA-ergic
Igtp - GABA-ergic
L2 Ngb - Glutamatergic
L2/3 Ptgs2 - Glutamatergic
L4 Ctxn3 - Glutamatergic
L4 Scnn1a - Glutamatergic
L4 Arf5 - Glutamatergic
L5a Hsd11b1 - Glutamatergic
L5a Tcerg1l - Glutamatergic
L5a Pde1c - Glutamatergic
L5a Batf3 - Glutamatergic
L5 Ucma - Glutamatergic
L5b Tph2 - Glutamatergic
L5b Cdh13 - Glutamatergic
L5 Chrna6 - Glutamatergic
L6a Mgp - Glutamatergic
L6a Sla - Glutamatergic
L6a Car12 - Glutamatergic
L6a Syt17 - Glutamatergic
L6b Serpinb11 - Glutamatergic
L6b Rgs12 - Glutamatergic
Gja1 - Astrocyte
Pdgfra - OPC
96_Rik - Oligodendrocyte
Opalin - Oligodendrocyte
Ctss - Microglia
Tbc1d4 - Endothelial
Myl9 - Endothelial
Htr3a, E18, type0 - GABA-ergic
Htr3a, P2, type1 - GABA-ergic
Htr3a, P2, type2 - GABA-ergic
Htr3a, P2, type3 - GABA-ergic
Htr3a, P5, type1 - GABA-ergic
Htr3a, P5, type2 - GABA-ergic
Htr3a, P5, type3 - GABA-ergic
(Lumbar) DRG neuron
(Precursor) Olfactory neuron
(Early Immature) Olfactory neuron
(Late Immature) Olfactory neuron
(Mature) Olfactory neuron
(Cochlear) Hair cell
(Vestibular) Hair cell
(Cochlear) Supporting cell
(Vestibular) Supporting cell
(Cochlear) Non-sensory cell
(Vestibular) Transitional epithelial
(E14.5) fibroblast
(2d) Ascl1 induced neuron
(5d) Ascl1 induced neuron
(20d) Ascl1 induced neuron
(22d) Ascl1 induced neuron
(22d) Ascl1,Brn2,Myt1l iNeuron
Cnih1
(ENSMUSG00000015759)
CT_03483
Exoc6b
(ENSMUSG00000033769)
CT_05400
CT_05399
CT_05398
CT_05397

Supplemental Figure 6. Alternative splicing analysis of full-length single-cell RNA-Seq data. As a proof of concept, we demonstrate that our splicing analysis method can detect alternative exons in single-cell RNA-Seq data. We used data generated from V1 mouse cortex and merged junction counts based on the 49 cell types identified in Tasic et al.[13] (**A**) Splicing analysis reveals a wide degree of variability in exon PSI that can differentiate not only between glia and neuron but also between individual subtypes of glutamatergic or GABA-ergic neurons. (**B**) Furthermore, we can robustly identify mutually exclusive exon splicing patterns used by different neuronal subtypes. (**C**) Analysis of SmartSeq2 single-cell data generated from E14, E18 and P2 Chx10:GFP-positive mouse retinal progenitor cells[14] reveals that even at early stages of development, we can begin to detect photoreceptor-specific exons. Rod-specific exons in *Atp1b2*, *Ttc8*, and *Slc4a7* are primarily detected in photoreceptor precursors. Neurogenic and amacrine cells also appear to utilize the exon in *Slc4a7* at low levels. In contrast, a pan-neuronal exon in *Sgce* is fully utilized in retinal ganglion cells and photoreceptor precursors but is only moderately incorporated in neurogenic cells. In addition to V1 cortex, we have also analyzed several other single-cell studies in the CellTower compilation of ASCOT (http://ascot.cs.jhu.edu) and can identify (**D**) the same cell type-specific exons in *Sptan1*, *Cnih1*, and *Exoc6* as we found in Fig. 1B-D. The exon in *Sptan1* is only found in cochlear/vestibular hair cells, the exon in *Cnih1* is primarily enriched in glutamatergic neurons, and the exon in Exoc6 is only detected in mature oligodendrocytes.

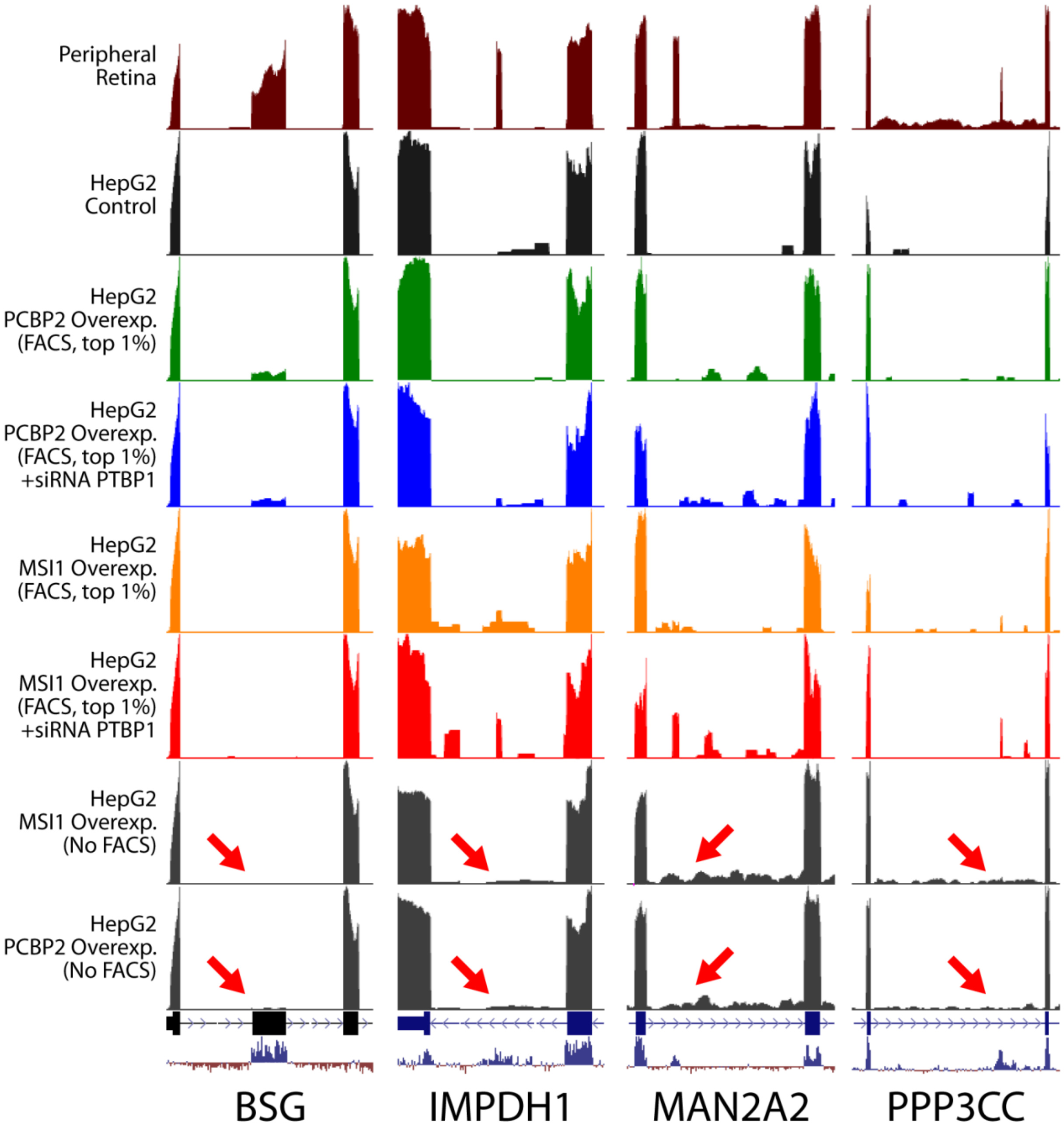

Supplemental Figure 7. Activation of rod-specific exons in HepG2 cells requires FACS isolation of the most strongly expressing cells

Normal transfection of MSI1 or PCBP2 is unable to strongly activate rod-specific exons in HepG2 (bottom two rows, red arrows). Rod-specific exon activation is only achieved after isolating the top expressing cells with FACS.

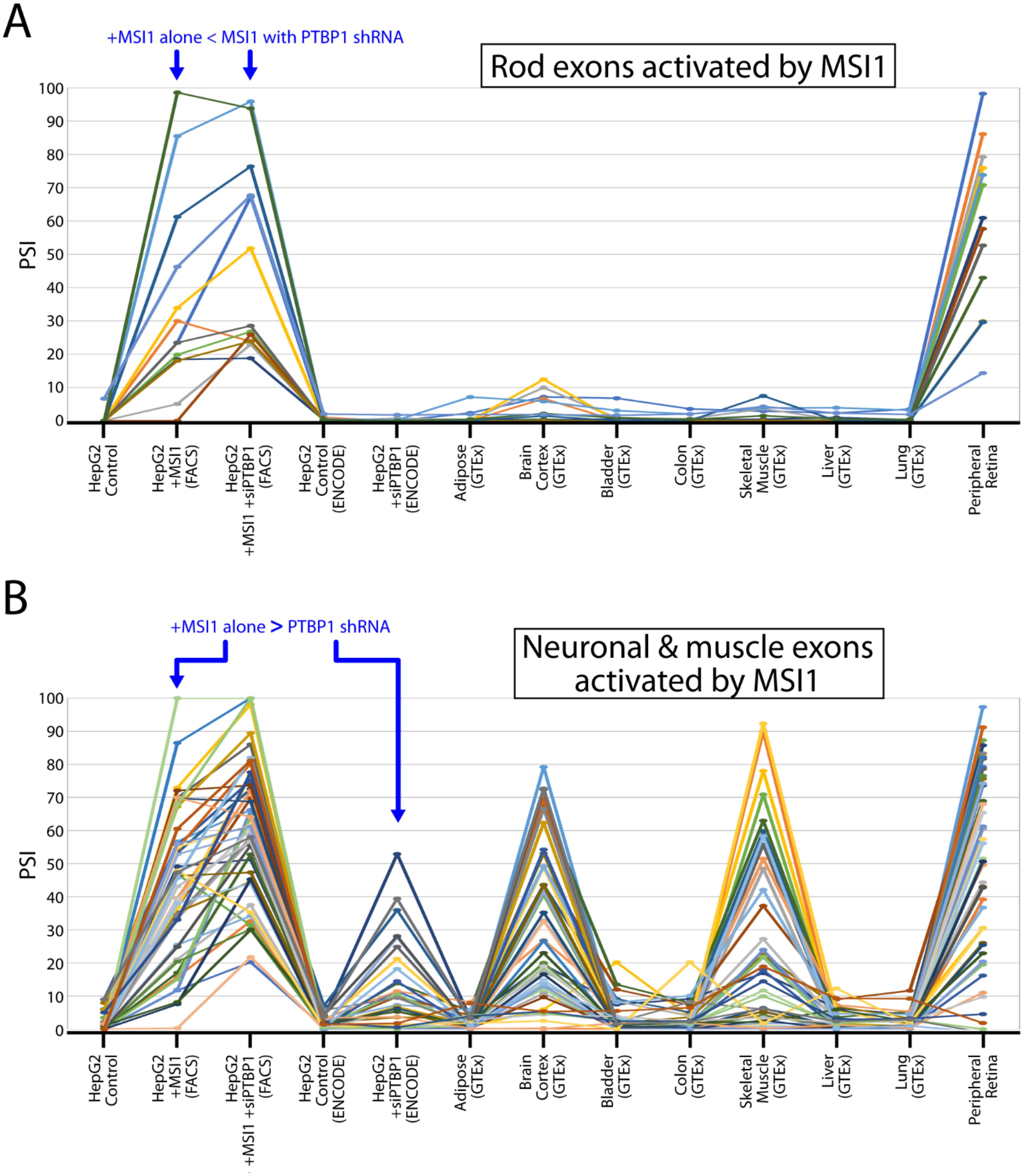
A
+MSI1 alone < MSI1 with PTBP1 shRNA
Rod exons activated by MSI1
PSI
100
90
80
70
60
50
40
30
20
10
0
HepG2 Control
HepG2 +MSI1 (FACS)
HepG2 +MSI1 +siPTBP1 (FACS)
HepG2 Control (ENCODE)
HepG2 +siPTBP1 (ENCODE)
Adipose (GTEx)
Brain Cortex (GTEx)
Bladder (GTEx)
Colon (GTEx)
Skeletal Muscle (GTEx)
Liver (GTEx)
Lung (GTEx)
Peripheral Retina
B
+MSI1 alone > PTBP1 shRNA
Neuronal & muscle exons activated by MSI1
PSI
100
90
80
70
60
50
40
30
20
10
0
HepG2 Control
HepG2 +MSI1 (FACS)
HepG2 +MSI1 +siPTBP1 (FACS)
HepG2 Control (ENCODE)
HepG2 +siPTBP1 (ENCODE)
Adipose (GTEx)
Brain Cortex (GTEx)
Bladder (GTEx)
Colon (GTEx)
Skeletal Muscle (GTEx)
Liver (GTEx)
Lung (GTEx)
Peripheral Retina

Supplemental Figure 8. Increased *MSI1* expression and *PTBP1* downregulation may interact synergistically to activate rod/neuron-specific alternative exons

Exons activated by *MSI1* overexpression in HepG2 cells can be grouped into two categories: rod-specific exons (**A**) and neuronal/muscle exons that are also regulated by *PTBP1* (**B**). Two lines of evidence suggest that *MSI1* interacts synergistically with downregulation of *PTBP1*. First, activation of rod exons is stronger when *MSI1* is combined with *PTBP1* knockdown (**A**). Second, overexpression of MSI1 alone can activate neuronal/muscle exons more effectively than *PTBP1* knockdown in HepG2 cells (**B**).

Upstream flanking sequences of rod-specific exons (-75 to +20 from 3’ splice site)

YELLOW = PTBP1/PTBP2 binding site (repetitive CU/UC)

3’SS ↓

(human)	ADGRV1	AUUUGCUUCUUUGUUGCUUUAUUGUGUUCUCUGCUUCUUUCAUGACCUUUUAUCUUUUUCUUUUCAUGCUUUUAGAAAGACUCAGUUAAUUCAU
(mouse)	Adgrv1	AGUUUAAUAUUUAUUUUGUGAAUUCAGUGCUUGCUUCUAUUCUGACCUUUGAUCUUUUUCUUUUCAUGCUUUUAGAAAACUUAAUUCAUUACAGA
(human)	AMPH	AUACAGUGAAGAAAAAGCGGGGCCAUGACCACUUAAUGAAUGUUCCUUUCACUUUUUUUCAACAUUCCUCUGCAGAAUGAGGUGGUGAGUGAGCU
(mouse)	Amph	AAAACAAGAAGUAGGGUUACAGCCACGUAACAAAUGUCUCUUAUUUUUAUUCCUUUUUAAAACAUUCCUCUGCAGAGCGAGGUGGUGAGUUAGCU
(human)	AMPH	UCAUCAAAUCUUACCAUGUUCUGUUUCAUCUUUUGUUUUUGUUUUUUUUCCUUUGCUCUUCUGCAUACUUUAAAGACUGACUCUUCAGAAAGUCU
(mouse)	Amph	GACCUAUAUGUUUCUCAUCAAAUAUCUCAUGGUCUAUUCCAUUUUUUCCUCUUUGCUUUUCUGCAUACUUUAAAGACUGACUCUUCAAAAAGUCU
(human)	ATP1B2_ex1	CUGAGUGGCUCACCUGAGUGUCCUUCAUGGUUUCUGUGUAAUUCACCUGUCUCUCCCUAUCUUCUUUGCUCCUAGAGGCCCCAUCACCAUAGAAA
(mouse)	Atp1b2_ex1	CUGUCCUCUCUAGAAACUCUCCUCCUUCCCACUGAGCCUUGACUGUUUAUCUGUUCUUUCUUGGCUCUGCUCCAGAGACUGAUUCCCAAGGACGG
(human)	ATP1B2_ex2	CUCCCUCCACAUCCCCUUCCUUGCUUCCUAUUCAACCCUUAAUCAUGUAUCUCUUCUUUCUUGGCUCUGCUCCAGAAACUGAUUCCUGAGGAUGG
(mouse)	Atp1b2_ex2	UUACCUGCCCACCUGGUUAGACUUCCUGGUUUCUGAGUGCUUCACCCAUAUCUCCCUAUCUUUUUGUGCUUUCAGAGGCCACAGCACUAUAGGGA
(human)	BSG	GCGAGGCCUUCCCCAGGAGCUGCCCUCCCAAGUGCCCCAGGCACUAACAAGACCCCACGCGUGCUCUCCCCACAGCCGGCUUCGUCCAGGCGCCG
(mouse)	Bsg	UGUUGUGUUGGCCCCUGUGGCAGGCCCUGGUGGCUGUCCUUCCUUGUAGUAACGGGUACUAACCCUUUCCCAUAGCUGGUUUCCUCAAGGCACCA
(human)	CASK	UAUUUUUCUUGAAUCAUCAUCUUUGCUUUGUUUUUGUUUUGCCUGUUUUCCUGCCCUUUUACUAAUUUUCCAUAGCUGCCAAAAUGGUGCAGAAA
(mouse)	Cask	UCUUGAAUUAUCAUCUUUGCUUUGUUUUUGUUUCUGUUUUGCCUGUUAUCCUACCUUUUUACUAAUUUUCCAUAGCUGCCAAAAUGGUGCAGAAA
(human)	CC2D2A	UUUUUUAAAGAUUAUCUCUGUUACUAAAGCCUGCCUCUUGUUUCUUUUUCUUUCUUUCUUUCUUGCCUCUGAUAGAUGAGUAACAUGCUUAAGAA
(mouse)	Cc2d2a	AUUAUUUUGGUUAAAAGCCUUUAAGCUGUUGCUAAUACUGUGUUUCUAUCAUUUCCUUUUUAUUGCCCAUGGUAGAUGAGUGACAUGCUUAAGAA
(human)	CLTC	UCACUGUGUAUAAAUGAAUUCCUGAUGGAAACUGUUGUUUUUUGUGCUCUACCUUAUGUCUGUGGAAUGUAACAGGACAUGUAUCAGGUAAAUGC
(mouse)	Cltc	UCACUGUGUAUAAAUGAAUUCCUGAUGGAAACUGUUGUUUUUUGUGCUGUACCUUUUGUCUGUGGAAUAUAACAGGACAUGUAUCAGGUAAUUGC
(human)	DMD	GGGGGACUUAGUGAGAUUAAAGACUUCUGAUUCACUUUGUAUUUGAAGAAUUUUUUUUCCUCCAUCUUUGCUCAGCUAGUGGAAUCCAUGAUGAA
(mouse)	Dmd	GGGGAUUUAGUAGGAUUAAAGAGUUCUGAUUCACUUUGUAUUUGAAGUUUUUUUUUUUCCUCCACCUUUGCUCAGCUAGUGGAAUCCAUGAUGAA
(human)	DOC2B	CAAGCAUGUGUCAGCUCCAUGUUCUCAACUGUCCUGUUCCCUUCCCCUCACCCCAUUGCUCCCUGUGGGAGGAAGGUGCCAAAAUUGAUGUUCUC
(mouse)	Doc2b	CUCUGCAUUGAACCUGCGUCUGUUCCAUGGUCUCAUCCACACCGUUCCCUUCACACUGCUCCCUCUGGGAGAAAGGUGCCAAAGCUGAUGUUUUC
(human)	EFR3A	AAGAUUAUUAGAGCUUUAUAAUUAAAAUAUUAAAAAAACAUUUAAAAUAUUUUACUCUUAUAAUUUGAAUUUAGCUCUCUUUCCUUCAAGCAAA
(mouse)	Efr3a	AUUUAAGGUUUUUUUUUGUUUGUUUUUUUUUUUAAAUGCAUUUAAAAGAUUAAAUAAUUUUACUUUGGAUUUAGCUGUCUUUUCUUCAGGCCCA
(human)	FARP2	GUUCAUAAAUGAAUUGUCCAACUCCCUUUUGGAUAAUCAAAAUGCUCAACAUUUUUUCCUUUGCUUGAUUUUCAGCUAAUAGUGCUGGACCCCCC
(mouse)	Farp2	AUGUUGGUCCCAGGGUGAUGAGUUUUCCAGCUCCUAGUACUGAAAUCCUCAACAUUUUCUUUGCUUGAUUUUCAGCUAAUAGUGUUGGACCCACC
(human)	GRAMD1B_ex1	UUUUGAUUUCUUGUUCUUCCUCUCCUCUUCUUCCCCACCAUUUCCCAAAUACCUUCUUUGCAUGCAUGGUCUUAGUCUCACAAGCGCCUCUCAAA
(mouse)	Gramd1b_ex1	CUGUGACCUUUCACCUUUUUGUUCGUUUGUUUAUUUGUUUGUUUGAAUUCUAAUUUUUGUGGUUUUUUUUUCCAGUAUGACAGACUUAAUCUGAU
(human)	GRAMD1B_ex2	CGCUGUGGCACUGACCCUUCUUUGACUUUCCUUUUUUUUUUUUUUCAAAUCUAAUUUUUGUGGUUUUCUUUCCAGUAUGACAGACUUAACCUGAU
(mouse)	Gramd1b_ex2	GAACUUGUCUUUUGAUUUCUUGUUCUUCCUCUUCUCCCCACUGUUUCCAAACACCUUUGCAUGCAUGGUCUUAGUCUCACAAGCGCCUCUCAAA
(human)	HCN1	UUCUUGAGAGAUCUGGGUCCCUAUCUAUCUCUUCUAAGUAUUCUGGGUUUAUUUAUUAAUGUCUAUUUUUGCUAGCUAAACAAACUGAUUUCCCG
(mouse)	Hcn1	GUAUAGAAAGAUCCGAGGCGGUACCUGCUCCUGCUAGGUGCUCUGGAUUGAUUUGUUCAUGUCUGUUUUCACUAGCUAUGCAAUCUGAUAACCCA
(human)	IFT140	UGUCUUGCACACACUCAUGCAUCUGUUCUUUUCUCUCUCAUUUCUCACCUUCCCGCAUGCGUCCACUCCUCAUAGUUGUUGUUCCAGAGAGCAGG
(mouse)	Ift140	UGCAUUUCUGUUUCAUCCACACAUUUAUGCAUGCUUUCUUUUUUCUCUGCUCCCUCUCUGUGCCUGCAUUCAUAGUUGUUAUUCCACAGAAGAGG
(human)	IMPDH1	CCCUGGUCAGGAGCCCUGUCCCCAGGUAUCAGCGGGCGUCUAACCUGUGUCUCUUUCCGCCCCACCCUUCCGUAGCUAUACCUUUCUGCCGUGUA
(mouse)	Impdh1	UGGUCGAGAGGACCCAUCUCCCUAAUCAUAGGCGGGCAUCUAACCUGUGUCUCUUUCUGCCCCACCCUCCCAUAGCUAUACCUUUCUGCCGUGUA
(human)	INTS13	AUUGUUGGACAUGUAUUAUUUAUUUUAAUUGAACCUUUACCUUUUCUCCCCAUAUCGUAUUUUCUGUUUUGCUAGCCCACAUUCAAUGUCUUUGA
(mouse)	Ints13	UGUUUGCUUGAUAUGCCUUAUUCACUUUAAUUGAACCUUUACUUUUUCUCCCCAUAUCAUAUUUUGUUUUGCUAGCCCACAUUCAGUGUCUUUGA
(human)	KCTD5	GGGCCCGGGGCACCCGCCCACCCGCACUGUCCUGACAGCCUGUCUUCUCCCCCACUGCGCGCUGCACGCCGCCAGAGUGACGACGAGGACCAGGG
(mouse)	Kctd5	AGUCCUCCUGCAGGCCUGCACUGCUUAGGCUGGGCUGACAGCUGUCUUCUUCCUUACUUCCUGCCUUCACACCAGAGUGACCAAGAGGAGCUGAG
(human)	KIF1B	AUUUUUUCUGAUUCUCUCCCUUAUUUCUUUUGUUUUUUUGCUGCACUAACUAAAGCCUUUUCUAUGUUUUUGCAGGCUCUUUCUUGAUAAGGAUA

(mouse)	Kif1b	GUAUAUAUUUGUUUGUUUUUCUUUCUGUCUGUCUUUUGGUUGCUGGACUAACGAAGGCAUUUUCUGUUUCUGCAGGCUCUUGCUUGAUAAGGAUA
(human)	KIF1B	CCUAUUCGCCAAAUUCCCACCCCACCCUGUGCUCACUCUCCACCCUCUGUAUUCCCCAUCUUCAUCUGCCUUCAGACCAUGAGAGAAAGAUUGAG
(mouse)	Kif1b	CCACUUACCAAAUCCCCACCCCUGCCCUGCACUCAUUCUCCUCCCUCUACCUUUCCCAUCCCCAUCGGCCUUCAGAGCAUGAAAGAAAGAUUGAG
(human)	KMT2D	UAAAUAGUUAGAGAUAUAAUAGAAAAAUAAUAAAACCUUACAUUUGUAUAGCACUUUAUACUUUUUUUUUCAAAGCGUUUUCACAUCCAUUAUCU
(mouse)	Kmt2d	UAAAUAACUAGAAGUAUUCUACAGAAGCACUGCAGCCUUCCGUUUGUAUAGCACUUUAUACUUUUUUUUUCAAAGCGUUUUCACAUCCAUUAUCU
(human)	MAN2A2	CCUCUGCCCUGUCCCAGCAUUCUCUAAGCCUGUGCUCCAACCCCGCUCAUCUCAUGGCUUUCUUUGCCCCACCAGCCUGACUUUUUCUCCAAACU
(mouse)	Man2a2	UUCUCACCCCAAGCCCGAGCACUUCCUAACCUUGACACAACACUGCUCAUCUCACAGUUUUCUUUGCCUCACCAGCCUGGCUUUUUCUCCAAACU
(human)	MARCH1	CAUUCUCUCUCUCUGUUUUUCCAUGGCUUUCCUCUCCAUUCCCAUUUCCCAUCCUUUGCCUGCUGCUCUGUCUAGAUCUGCCAUCUUGCAUGACA
(mouse)	March1	UCUUUCUCUCUUUGUUUUCCCAUGACUUUCCUUUCCAUUCCCACUUCCCAUCCUUUUGCCUGUUGCCUUGUAUAGAUCUUCACUCUUGCAUGACA
(human)	MSI2	GGAGCUUGGAGACCUGCAGCCAGGGACAACCAUACAUUUAUUUCACCUGGAUCUUUCUUUUUCCCCCUUCAACAGCCCUAUUACCAUAUUUAAAU
(mouse)	Msi2	UGGGAGACUCAUUCCCAGCCUAGGAGAACCGUGUACAAUUGUUUCACCUGGAUCUUUCUUUUUCCCCUUCAACAGCCCUAUUACCAUAUUUAAAU
(human)	PLEKHB1	AGUCUCUUUAGGUGUAGAGGGCCUCUGACACCCCCUCCCUCCCUCCUCCUUCUUGGGUUUCUGUGGCUUGAGCAGGCCCCAGCUGGAGCCACCGU
(mouse)	Plekhb1	CCUAGUCUCUUAAGAUGUAAAGGGCAUCUGACAUCUCCCUCCCUCUCCCUUCUUGGGUUUCUGUGGCUGGAGCAGGCCCCAGCUGGAGCCACCGU
(human)	PPP3CC	CCAUGUAAACAUAAAUUUCUCUUCUCAUAUUUCUGUCUUCAUCUCCUUGUUUCUUGGUGUUUCUUCUCCCUGAAGAUCACUACAUUCCAAGCUAU
(mouse)	Ppp3cc	UCCAUGUAAACUCCCAUCAUCCUCCCAUGUGUCUGUCUUCAUCUCUUUAUUUCUUGGUGUCUCUUCUCCCUGAAGAUCACUACAUUUCAAGCUGU
(human)	SLC4A7	UGUUCCUCUGUCACAGUCUUACUUUUUUUUACUUGGCCUAUCUUUGUGUAUACUUCCUUGUCUCCUGUUGGGCAGGAGUCUGCCUCCUGGCACUG
(mouse)	Slc4a7	UUUUGUUCCUCUGUCACAGUUUUACUUUUUUACUUGGCCUAUUCAUGCAUACCCUCCCUGUCUCUUGUUGGGCAGGAGUCAGCCUCCUGGCACUG
(human)	STXBP5	GAUUGUUGUCCCAACCAUACCAUUCUAGUAUUUUUGUCUCCUUUUAUUUCUUCCGCUUUUCAUUGCUUCACUAAGGUGUGCUGAUUGUGACUUCU
(mouse)	Stxbp5	CAGCAGAAGCUCUGUUCCAACCAUGCCAUCUAGUGUCUGUCUCGUUUCUCUCCCUCCCCCUCAUGGCUCACUGAGGUAUGCUGCUUGGGACUCCU
(human)	TTC8	GGUUGGUCCUUAGGACUUUUUAUUUUAGAAACUGUAUUUCUUAAAACUGUUUACUGCCUUCUUAAUGCUUUCCAGGAACCAGAUCCUGAAUUGCC
(mouse)	Ttc8	UAAUACUCAUUAAGACUUUUGUCUUCAGAAAUUGCAUGCCUUAAAAAUGUUUACAACCUUCUUAACGCUUUCCAGGAACCAGCUCCUGAUUUGCC
(human)	UNC13B	GUCCCUGAGGGUCUCUCUGAUGUCCCUGAUUUUUUCACUCUCUUUGUUGCUCAUUGGUGUCUGCUCUGUCACAAGCAACCACUGUCAUUCAUAUA
(mouse)	Unc13b	AAUAGAAAGGAGGGUCUCUGGCAUCCCCGAUUUUCACACUUUUUGUUGCUCAUUGGUAUCUCUGUUUGUCACAAGCCACUUCUAUCAUUCGUAUU
(human)	UNC13B	CUUCUUUCUUCCUUUUCCCUGUCUCUUCCUUUUUCUUUGCUUUAUUGUUCCUCCAAUUUUAUCCCUUCACAUCAGACAAGGCUCACAGGCUAAAG
(mouse)	Unc13b	AACUCAUUUUUUCCUUUUCUCUGUUUCUCCUUUUUUUUUUUUCCAUUUUCUUUAUUCCUCUAAUUUUAACAUCAGACAAGACUGGCAGGCUAGGA

Downstream flanking sequences of rod-specific exons (-20 to +75 from 5’ splice site)

RED = MSI1 binding site (UAG clusters, <15bp apart)

5’SS ↓

(human)	ADGRV1	AAUUCAUUACAGACUGAAAG	GUAAAACCUGAGCCUUGGUACAAACUUAUCUAGAUUCCUUUGAAAGAUGUAUUCCUAUGUUUAAACACAAACUC
(mouse)	Adgrv1	AAUUCAUUACAGACUGAAAG	GUAAAACAUAACUUGGUAGAAACUUAAUUAGACCCUUCAAUAGAUAAAGUCUAAGUAUUGAUGGAGAGUGUGUGA
(human)	AMPH	UUCCUCUGCAGAAUGAGGUG	GUGAGUGAGCUCAGUGGCAUCUCUAUUAAGCUUAGAUUGGGGCUAUGUUUCCAUCUGAGGCCAUGAUUGCUGGCA
(mouse)	Amph	UUCCUCUGCAGAGCGAGGUG	GUGAGUUAGCUCUGUGGGCAAUCUCUAGACUAGAGCAUAGACCGAGCUGUUUCUAGUCAGAGGUCAUGAUUGCUG
(human)	AMPH	UAGAGGCUGACAAAGACGAG	GUACUUACCCUGGUCUGUAGCAUAGACUAGCCAAAGACUUCUGUAGGCUCUGUUUUUCCUCAUGUCUGUCACCUA
(mouse)	Amph	AGUUAGAAGAUCCUAUGUGG	GUAUGUGUGGAGACAUACCAAAGGGUAGACACUGGAGCUAUUGGUAUCAAGGCAAUAGAAGGUACUGAGGGAUUU
(human)	ATP1B2_ex1	AUCACCAUAGAAACAAGGGG	GUAAGAGUGGGCUUUUGGGCUCCACUGUAGCUUGAACUCCGAGGGCCCCGCACUCCUCUUGCUUCUCUCUGGGAU
(mouse)	Atp1b2_ex1	GACUGAUUCCCAAGGACGGG	GUAAGAACUUGGGGAUUGAUGGUGGAGUUAGAAGGCCCUCACCGUGUUGUCAGCACCCUUAGAAGACCUAGUCUG
(human)	ATP1B2_ex2	AACUGAUUCCUGAGGAUGGG	GUAAGAACUUGGGGUAGGAGUGGAGUAGGAGGCUUUCGUGCCAUUAGCAGCCUUCAGGAGUUCCUAGAAUGAUGA
(mouse)	Atp1b2_ex2	AGCACUAUAGGGACAAGGGG	GUAAGAGUGGGCGCCUAUGCAGUUUUAGCUCUAAGAGGCUCUUAGCCCUAUUGCUUCUCUCUAGGAUAAAGAGAG
(human)	BSG	AGUCGUGCUAGUCCUGGAAC	GUGAGUGGCGGGCACCUCCCUCCCCGCCUCCCUCAGUUUCCCUCCUGUGCCGCUCGCCUCCCGGCUCCUGCUCAG
(mouse)	Bsg	GAGCGUGGUGGUCCUUGAAC	GUGAGUGGCCAGGGCACCUGUUUCUGUUUACCUCUCGUGCCACCCAACCCUGGCUCCCACUUUGUAGAACCAGAU
(human)	CASK	AAUGGUGCAGAAAAUUGCCU	GUAAGUACCAGCUAGGAGGUGGUGAAAUACAGCCGUGUUUCUUUUAAUAGAUUUAGAAUGUAGACACUUUGUUCC
(mouse)	Cask	AAUGGUGCAGAAAAUUGCCU	GUAAGUACCAGCUAGGAGGUGAUGAAAUACAGUCUGUGUCUUUUAAUAGAUGUAGAAUGUAGACAUUUGGUUCCC
(human)	CC2D2A	UGAGUAACAUGCUUAAGAAG	GUACUAAGUAUUUUGUUUUACUAUUUUAUUGGUCAAUAGUUAUUAGGCUUUAAUUAGCUGCUUCUGUCCACUGCC
(mouse)	Cc2d2a	UGAGUGACAUGCUUAAGAAG	GUACCGAGCAUCUUGUUUUAUAAUCCAGCAGUCAUCAGUUAAACGCUUUUACUAGCUGCUUUUGUUUACUGCCUC
(human)	CLTC	UGUAACAGGACAUGUAUCAG	GUAAAUGCCCAGUACAGGUUUUACUUACCUGUCUAUAACAUCUUCUAUGUCAGUCUAUAAGUAGGAUUGUAGUUU
(mouse)	Cltc	UAUAACAGGACAUGUAUCAG	GUAAUUGCCCCACACAGGUCUUACCCUUACCUGCCUGUCUAUAACAUCUCUGUCAGUCUAUAAGUAGGACUCUAU
(human)	DMD	UGAAUUCUCAUCUCCAAGGG	GUAAGCAGUUUUUAGUAAAGCCCAGUAGCUGACUUAUGACUCCUUAGAAAUAGCAUUGAUUCCUUCCUUCUCCUG
(mouse)	Dmd	UGAAUUCUCAUCUCCAAGGG	GUAAGCUGUUUGUAGUAAAGCCUGAUAGCUGCCUUAGGUCUCUUUAGAAGUAGCAUCGAUUCCUUCCUUUUCCUG
(human)	DOC2B	AAUUGAUGUUCUCUCGCAAG	GUAAGGGCCUGCAUGGCCAGUAGAGUCUCCCUAGUAGUGCCAUGUAAGGGCCUGAGCCAGCCCAGCUCAGAUCCA
(mouse)	Doc2b	AGCUGAUGUUUUCCAGAAAG	GUAAGGGCCCGUGUGGCCAUUAGAGAGCCUUGUAGGACUGUGGAAAGGCCUGGGCCAGCCCAGCACACAUGGAAU
(human)	EFR3A	ACCCAGGCACAAGCACAAAG	GUAAAUCCAGUUGUCAGUUGGCCUGCCUUUUCCCUUACCUGUCCUCUAGGAAAAUAGCUUAAUAAAAUGUGCCU
(mouse)	Efr3a	ACCCAAGCAGAAGUACAAAG	GUAACUAGCUAUAUAGCUCCCCCUGGUGUCGCCUGCCCGUCCUGUAGGGAAAGCACUCCCUGGAUGCAGUGUUUA
(human)	FARP2	UAAUAGUGCUGGACCCCCCG	GUGAGUAUUUGGGUGCCCUCUGGCCCAUGGCUCACCCCCUGGGGUGCAGCUGCCAUGGCGCAGGGAUACCAGUGU
(mouse)	Farp2	UAAUAGUGUUGGACCCACCG	GUGAGUAUUUCUGUGUCUCUAGACCAGACCUCAGUCACUUGGCAGCAGUUGCCAUAAUGUAGGGACACAAGUGUG
(human)	GRAMD1B_ex1	CUCACAAGCGCCUCUCAAAA	GUAAGCCAUCUUUUGUCUCUGUCCUUUUAUCUCCCCUCUAGUGUCGAUCUGUCUGUAGUGCUCAGCACCCCCUCC
(mouse)	Gramd1b_ex1	ACAGACUUAAUCUGAUCAAA	GUAAGACAUUGUUUUGUUUUGUUUUGAUUUUUUUUUGCCUCCCGGCUCCAGUUUAUGCUUAAUGUAGAGCUCCU
(human)	GRAMD1B_ex2	ACAGACUUAACCUGAUCAAA	GUAAGAAUUGUUUUUUAUUUCUAACUCCUACUAAAUGUAGAGCUCUCCUAGUCCCUGCCCCCCUACCUCCUCCAC
(mouse)	Gramd1b_ex2	CUCACAAGCGCCUCUCAAAA	GUAAGCCAUCUUUUGUCUCUGUCUUUUAAUCUCCCCUCUAGUAUUGAAUUGUCUGUAGUGCUCAGCAUCCCUCCU
(human)	HCN1	AAAACAAUUUGAAUCUUCAG	GUAAAAAGCAAUGAUAAAAAUUCUAGAUAGCAUUGAAAUAGAACUAAAAAUAUGAUAUAUGAGUCUAUAUUACAA
(mouse)	Hcn1	ACUUCAGUUUGAAUCUUCAG	GUAAAAAGCACUGGUAUAAACUCUAGAUAGCACUAAAAUAGUUCUUAAUGCUAAUAUCACGAGUCUAUAUUACAU
(human)	IFT140	CAGGCGUGUAUGACGCAGUC	GUAAGUACAGACGCCCAGGUCGUGUGUCAUUUGCUUCACCGGGUUGUUUUCCUGAGAUCCUUUUCCUCCCUUAUU
(mouse)	Ift140	GAGGUGUCUAUGGUGCAGUC	GUAAGUAUGAAGCCCCAGGCGUGGCUUGUCACCUUGGUUUUGUCAUGUCCCUGAGAUGUCUUUCUUCUCAUUGUG
(human)	IMPDH1	UAGCUAUACCUUUCUGCCGU	GUAAGUAACCGCGCUGCCCGGGCCAAGCGUAGAGUAGAGCAGGGGCAGUCUGGGUGGGACCUGGGCUGGAGGUGG
(mouse)	Impdh1	UAGCUAUACCUUUCUGCCGU	GUAAGUAACUGCGCUGCCGGGGCCAGGUGUAGAAUAGAGCAGGGGCAGUCUGGGUGGGCCAUGGGCUGGGAGAAU
(human)	INTS13	CAUUCAAUGUCUUUGACCAG	GUCAGUAAAUACUGAAAAGCAACCAGAAAGGAUCACAGAAUAAAAUGCUAUAUUUUUGUUUUCUAAUAAGAACAC
(mouse)	Ints13	CAUUCAGUGUCUUUGAACAG	GUCAGUAAAUACUGAAAAUCAAAAAGAAAGGAUCACAGACCAAAGUAUAUUAUUACUAUUUCUAAUAAGAACAUA
(human)	KCTD5	GAUUAAGUGUGAAUGUCAUG	GUGAGCUCCGUGGCCUCCUUGACCCCAUGGCCCCAGCUGACCCUUGCCACAGACCCUCCCUGUGUGUCUGUCCCU
(mouse)	Kctd5	GAUUAAGCAUAAAUGUCACG	GUGAGCUCUGCAGCCCUUUAAGUAGGCCUAGCACCAUAGUCUCCUUCCCAUAUGACCCUGGUGACUUUUGCCUGC
(human)	KIF1B	CUUGAUAAGGAUAUUCCUAG	GUAUUGUCUGUUUGCUUUUUUGCACGUAGGAUUCUAGUUUAUCAGAAUGCUUUCUGUCUUAUGUUGCUUUGCCAU

```
(mouse) Kif1b    CUUGAUAAGGAUAUUCCUAGGUACUUUCUGUUUGCUUUUUUUUUUUUUUUUUUUUUUUAAUUUAUUUAUUAAUUAUAUGUAAGUACACUGUAGCUG
(human) KIF1B    GAGGGUACCCAAACGAGAUGGUUAGUAGCUGAAUUAGCUCCACCUUAGAUGUAGAACCAUCCUCAGGCGCUUGUCCUGAAGCCUGGCUCUCUGCA
(mouse) Kif1b    GAGGGUACCCAAGCAAUAUGGUUAGUAGCUAAACUAGCCCUGCCUUAGAUGUAGAACCAACUUGGACAUAUGUGAAGCACUGAUGUCUCUGUUUG
(human) KMT2D    CCUCACAACAACCUUAUGAGGUAGGUAGGGCAGGUGGUAUGACCCCCAUUUUACAGCUGAGAAAACUGAGACCCAGAAAGAUUAAAUGACUAGGC
(mouse) Kmt2d    CCUCACAACAACCUUAUGAGGUAGGUAGGGCAGGUGGUAUGAUCCCAUUUUAUAGCUGAGAAAACUGAGUCCCAGAAAGACUGUGUGACUAGAUA
(human) MAN2A2   GCAGGAGCGGUAACCGAGAGGUAAGGGUCGGCUCUGGGAGCGGGGAACUAGUACGAGCAAGGUGUAGCUGCGCCAGAGCCCAGGUGACAGAGCCC
(mouse) Man2a2   ACACAAUCAGGAGCUCAAAGGUAAGGGCCAGGGAGGGCCUUGGUGGCCUAGGGCGAGCGGCCUGGCACUGUAGUUGGAGUCUGCUGCCUAGAACC
(human) MARCH1   GAUAAUUUAGAAGUAUGCAGGUCAGUCACUGUAGAGAAAGGCUUGAUGUCAUCCAUAUAUAGUUCUUUUUGCAGUGAGUUAGGUGCGUGUUUGAC
(mouse) March1   GAUAAUUUCGAAGUGUGCAGGUCAGUAGCUGUAGUGAAAGACUGGACGCAACCCAAUUAUAAGUCUUCUGGAUGGGAGCUCAGUAUGUGUUUGUG
(human) MSI2     AUUACCAUAUUUAAAUGCAAGUAUGUCUCUUUUCAUUGACAUUUUAAGUAGCUUAGCCUGACCUUUUUAGAGUAGCAUGUUCCCCCUUUGAACUU
(mouse) Msi2     AUUACCAUAUUUAAAUGCAAGUAUGUCUCUUUUCAUUGGCAUUUUCAGUAGCUUAGCCUGACCUUUUUAGAGUAGCUCUUUGCCUUUUGACCGUC
(human) PLEKHB1  AGGUUGAGAGGCGGAUCUGGGUAAGUGCUGGCUCGGCCCUCCCUGCCUCCAUACUGGCCACCAGGAUGAGCCUCCAAAACCAGGCCCAGUCCAUC
(mouse) Plekhb1  AGGUUGAGAGGCGGAUCUGGGUAAGUUCAAGCUCAGCCCUUCCUGCCUCCUCUCUGACCAUCAGGAUGAGCCUCCAAAGCCACCUGUCCCAUUCC
(human) PPP3CC   CAUUCCAAGCUAUCAGAAAGGUAUCCGACUAAACUGUGCUAAGAGCUUAAUGCUUACUCUCUGUCCCACCAUAGUCUAUUGGUUAGACUUAAAAU
(mouse) Ppp3cc   CAUUUCAAGCUGUCCAAAAGGUAUCCAACUGGAAUGCACUGCCAGCUAAGUUCUUGCUGUCUGCUCUGCCACUGGUGAUUGGUUAGUCGUCUUGA
(human) SLC4A7   UGGGACUCAAGAGGCCCGCUGUAAGUGCUGGUUUUACUUUUCAUAGAUAGCCCUGUAAGUGGUUGUUUAGUGACUUGUGUGAAACUGCAUAUAAA
(mouse) Slc4a7   UGGGACUCAAGAAGCCGGCUGUAAGUGCUGGUUUUAUCUUUUAGUAGAUAGUGUUGUUAGUGGUUGUUUAGUAGUUUGUAUGAGCUGUGUAUGAA
(human) STXBP5   ACUACGUUCUUCUUUUUUAAGUAAGUCAUUUAUGCCUUGGCAUGUUUGUUCUUUGUAAAAUCAUUGGGAGUUUUUGUGUUCAUUUUUAAAAGAGC
(mouse) Stxbp5   ACUCCGCUCUUCCGUCAUAAGUAAGUCAUUUCUGCCUUGAUACUUUUGUUCUUUGUAAAAUCACUGCUGCCUUUUUAUUUUACUUACUAAAAUAC
(human) TTC8     CUGAAUUGCCAGUGCAUCAGGUAAAGAAAGGUUUAGCUGCAACCUUUAGUUUAGAGAAUUCUUUAAUGCUAUUGCUUAGUUGUUGCUAUAGAUUA
(mouse) Ttc8     CUGAUUUGCCAGUGUCUCAGGUAAAAAGUUAGCUGCAAACUAUGGCUUAGAGAGUAGCUUAGUUGCUGUCGCUUAUUUGCUCCUGAAGAUUGGAA
(human) UNC13B   AAAAGUCCUCUUUCUGGGUGGUAAGAACAUUUAGUUGUCCUUUUGCCUCUAGUCUUACACCUUUUCCCUCUUCUCCUGCUCACUGCUUCAUAGAA
(mouse) Unc13b   UCGAAAAGUCUUCUUUGGUGGUAAGAACUUUUGGUUGUCCCUUUUGCCUCUAGUCUUAUAUCCUUCCUUCUCUUGCUCACUGCUUUCUAGAAGAG
(human) UNC13B   GAGCCCAUGACCAACAAAAGGCCUGUUCUUAACUCAAGCAUCAUAAAUGGCUUUAAGGAAUUGACCAUUUAUGAUGCAAGUGACUUAGUGGUAGA
(mouse) Unc13b   GAAACAGUAACCAACAAAAGGCCGGUUCUCCACUCAGGCAUCAUAAAUGGUUUUCAGGAAUUGACCAUUUAUGAUGCAAUUGAUUAGUAGUAGAU
```

Supplemental Figure 9. Upstream and downstream flanking sequences for rod-specific exons

Motif analysis of the sequences flanking the 31 rod-specific splicing events reveals motifs for both *PTBP1*/*PTBP2* and *MSI1*. We have previously shown that *PTBP1* and *PTBP2* bind to repetitive CU/UC elements upstream of the 3' splice site[15,16]. Previous work has also shown that *MSI1* preferentially binds to UAG motifs[17–19]. We observe that *MSI1* overexpression and *PTBP1* downregulation act synergistically (Fig. S9). Binding motifs for *PTBP1* (upstream) and *MSI1* (downstream) often flank the same exon, suggesting that *PTBP1* and *MSI1* may directly interact.

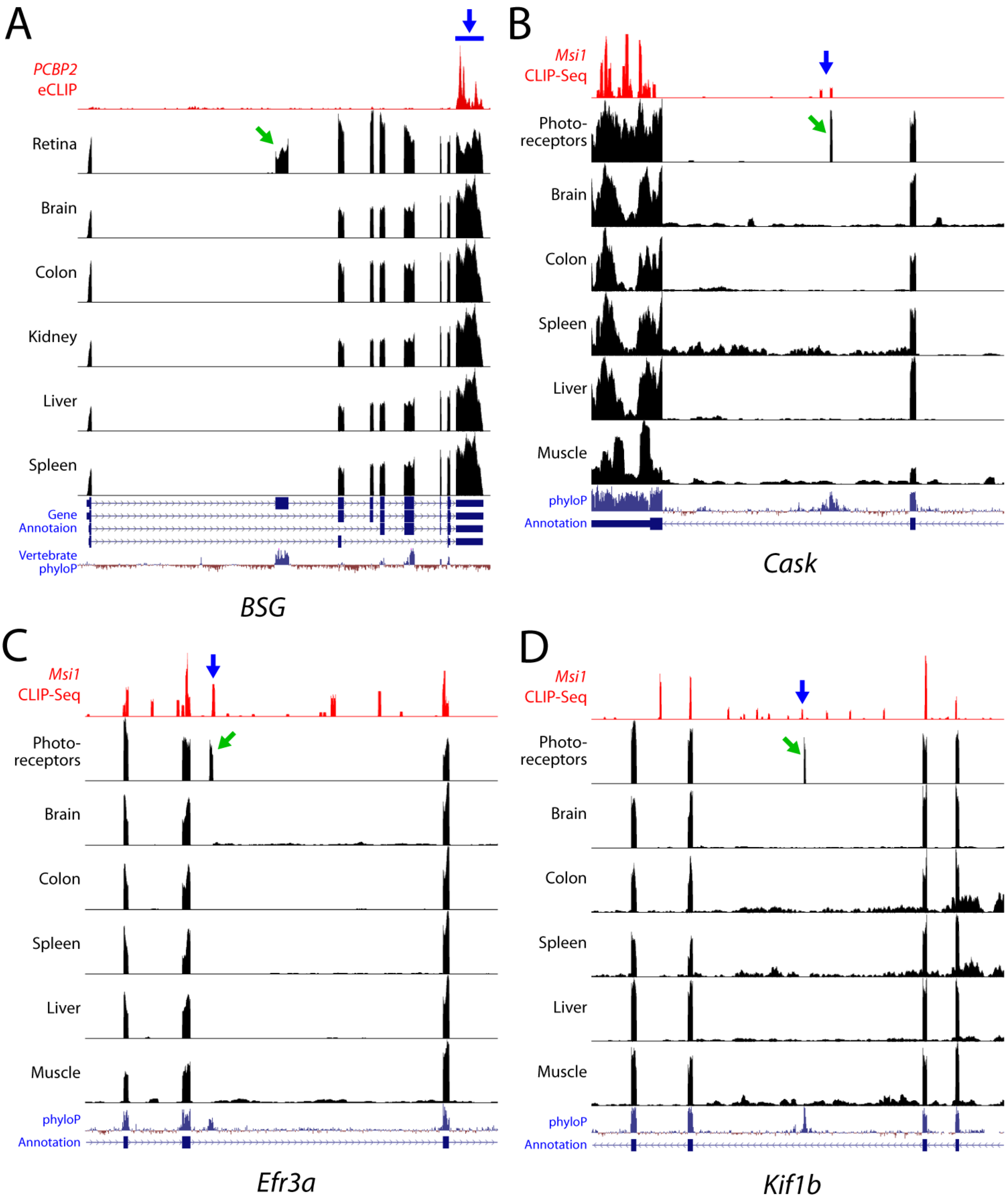
A
PCBP2
eCLIP
Retina
Brain
Colon
Kidney
Liver
Spleen
Gene
Annotaion
Vertebrate
phyloP
BSG
B
Msi1
CLIP-Seq
Photo-
receptors
Brain
Colon
Spleen
Liver
Muscle
phyloP
Annotation
Cask
C
Msi1
CLIP-Seq
Photo-
receptors
Brain
Colon
Spleen
Liver
Muscle
phyloP
Annotation
Efr3a
D
Msi1
CLIP-Seq
Photo-
receptors
Brain
Colon
Spleen
Liver
Muscle
phyloP
Annotation
Kif1b

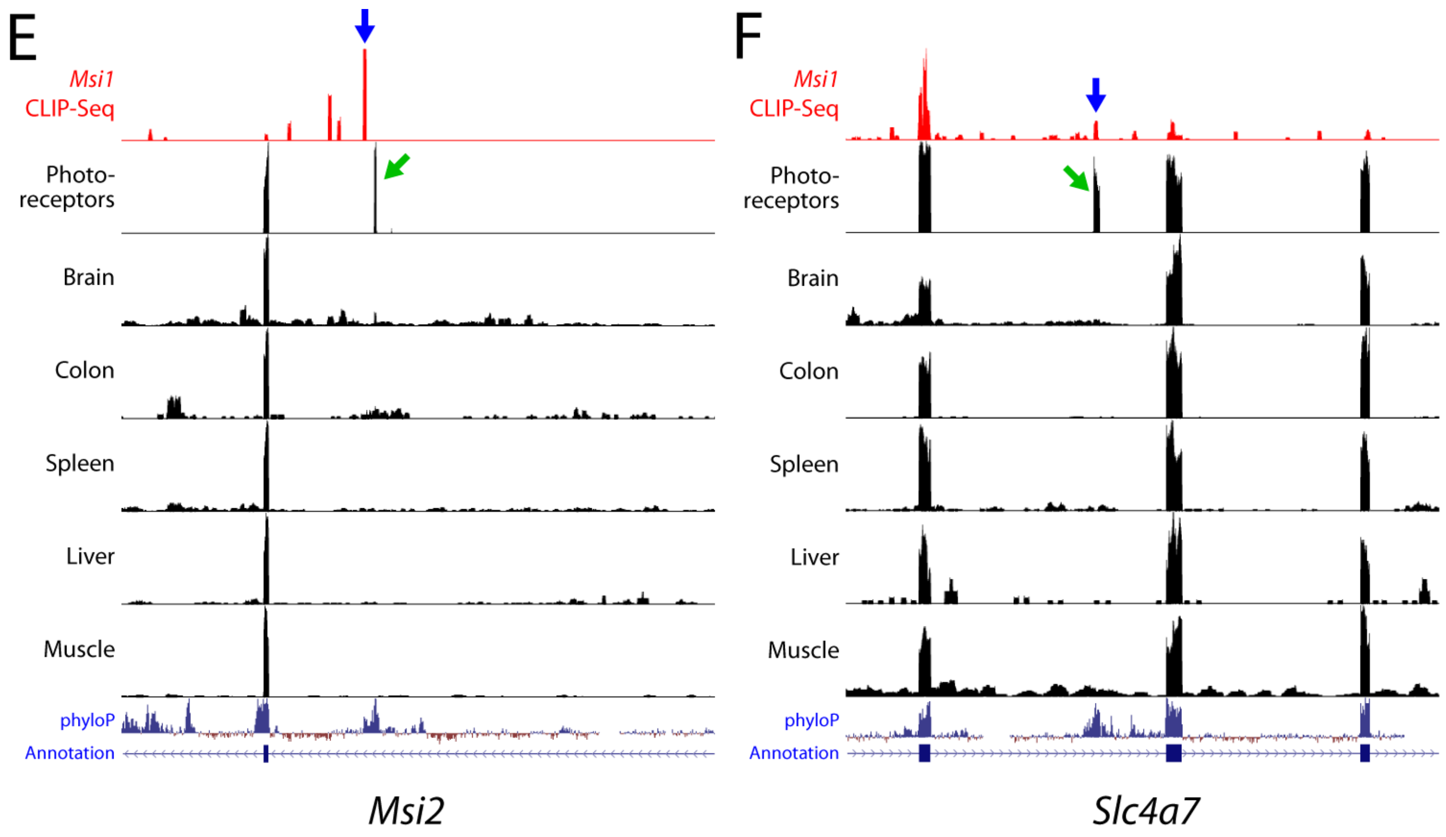

Supplemental Figure 10. *Msi1* CLIP-Seq supports direct interaction with photoreceptor-specific exons

(**A**) Analysis of PCBP2 eCLIP data from the ENCODE Project[20] indicates that PCBP2 does not directly interact with the photoreceptor-specific exon in BSG. However, PCBP2 interacts strongly with the 3'UTR of BSG, suggesting a possible indirect mechanism of action. (**B**-**F**) Using previously published Msi1 CLIP-Seq data generated in intestinal epithelium[21], we identify several exons with Msi1 CLIP peaks. Given that intestinal epithelium do not splice-in photoreceptor-specific exons and do not express many photoreceptor-specific genes, the presence of Msi1 CLIP peaks near photoreceptor-specific exons is significant. These intronic sequences are spliced-out and degraded and should therefore be underrepresented in CLIP-Seq data.

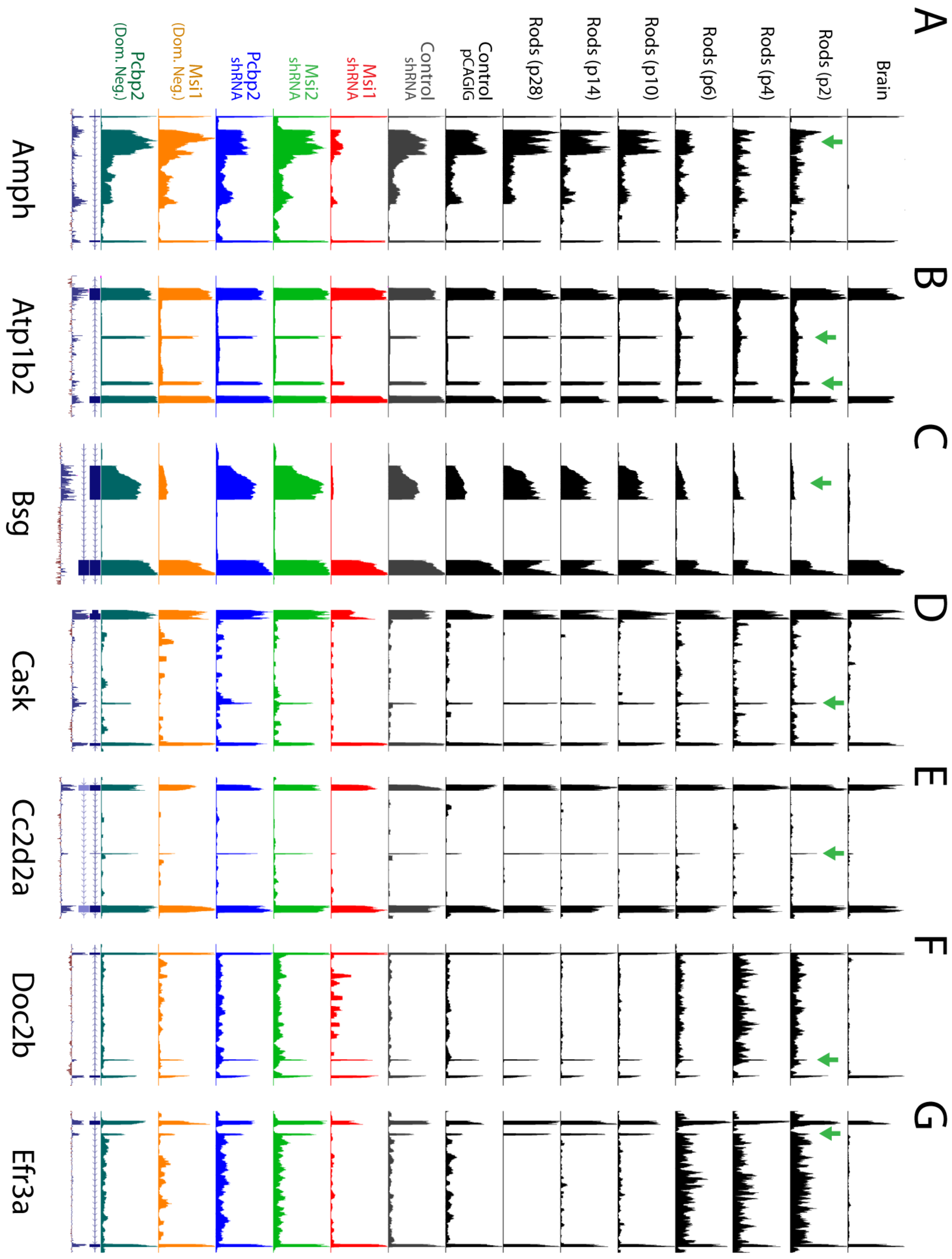
A
B
C
D
E
F
G
Brain
Rods (p2)
Rods (p4)
Rods (p6)
Rods (p10)
Rods (p14)
Rods (p28)
Control pCAGIG
Control shRNA
Msi1 shRNA
Msi2 shRNA
Pcbp2 shRNA
Msi1 (Dom. Neg.)
Pcbp2 (Dom. Neg.)
Amph
Atp1b2
Bsg
Cask
Cc2d2a
Doc2b
Efr3a

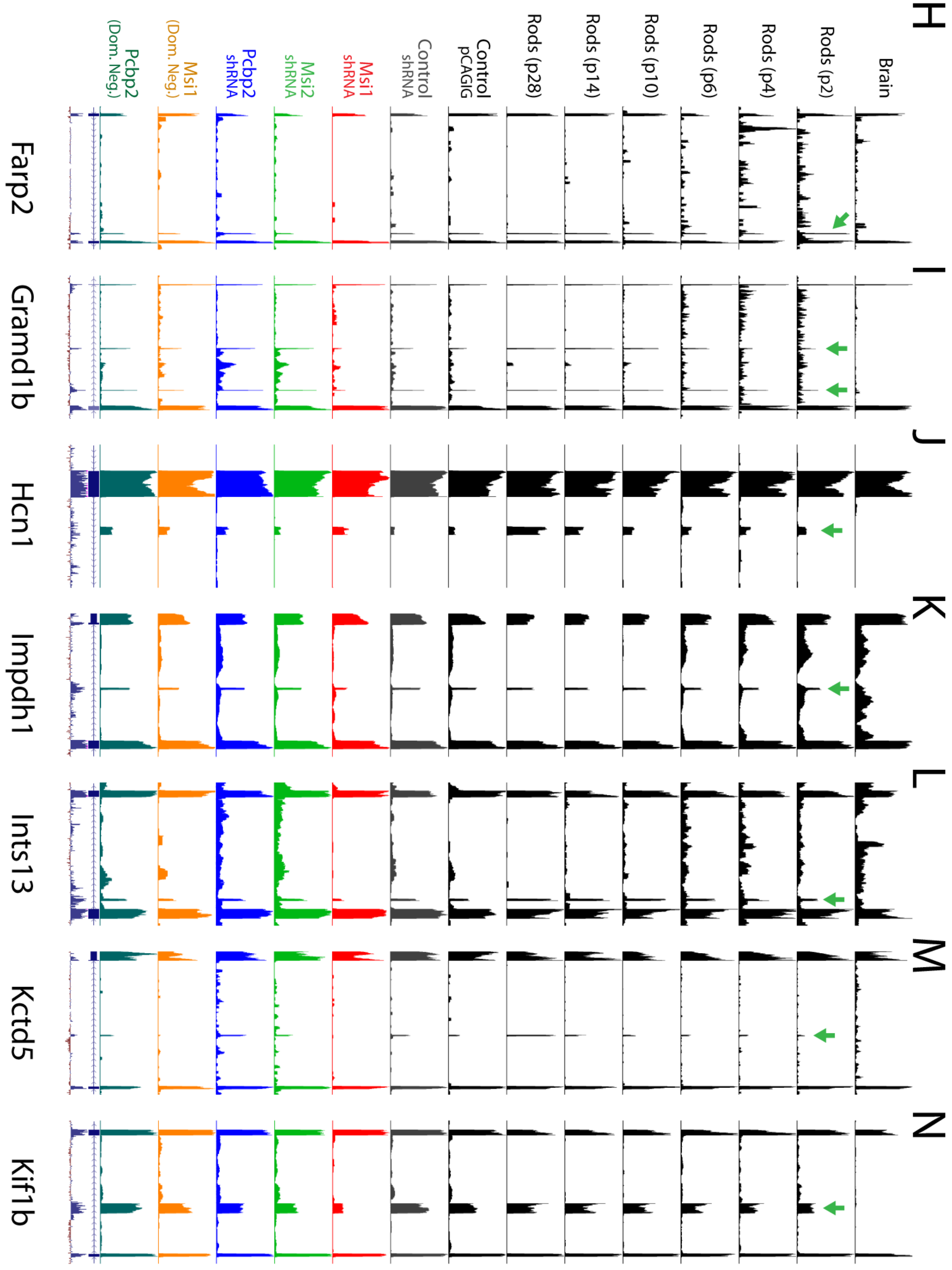

H
I
J
K
L
M
N
Brain
Rods (p2)
Rods (p4)
Rods (p6)
Rods (p10)
Rods (p14)
Rods (p28)
Control pCAGIG
Control shRNA
Msi1 shRNA
Msi2 shRNA
Pcbp2 shRNA
Msi1 (Dom. Neg.)
Pcbp2 (Dom. Neg.)
Farp2
Gramd1b
Hcn1
Impdh1
Ints13
Kctd5
Kif1b

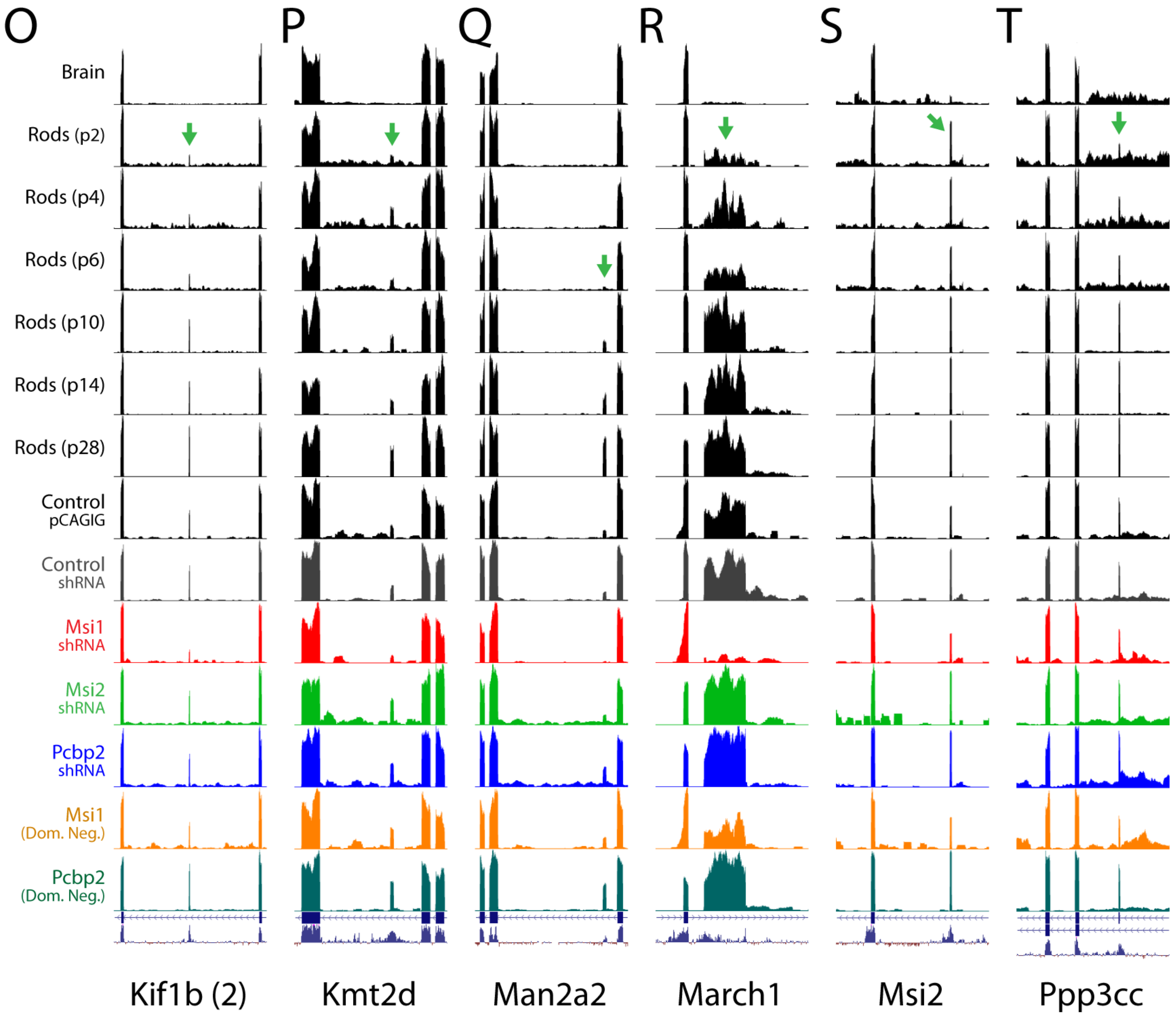

O
P
Q
R
S
T
Brain
Rods (p2)
Rods (p4)
Rods (p6)
Rods (p10)
Rods (p14)
Rods (p28)
Control pCAGIG
Control shRNA
Msi1 shRNA
Msi2 shRNA
Pcbp2 shRNA
Msi1 (Dom. Neg.)
Pcbp2 (Dom. Neg.)
Kif1b (2)
Kmt2d
Man2a2
March1
Msi2
Ppp3cc

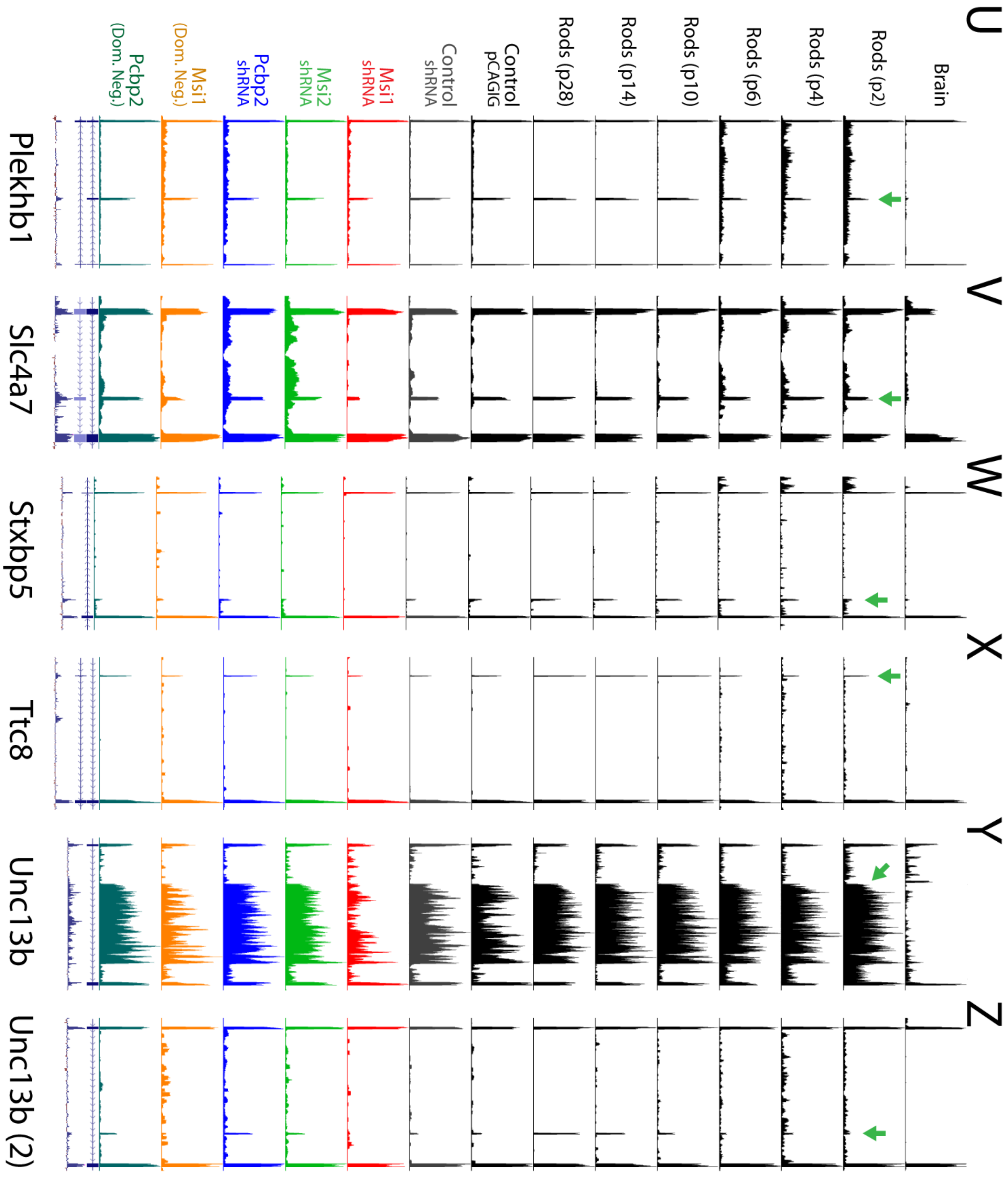
U
V
W
X
Y
Z
Brain
Rods (p2)
Rods (p4)
Rods (p6)
Rods (p10)
Rods (p14)
Rods (p28)
Control pCAGIG
Control shRNA
Msi1 shRNA
Msi2 shRNA
Pcbp2 shRNA
Msi1 (Dom. Neg.)
Pcbp2 (Dom. Neg.)
Plekhb1
Slc4a7
Stxbp5
Ttc8
Unc13b
Unc13b (2)

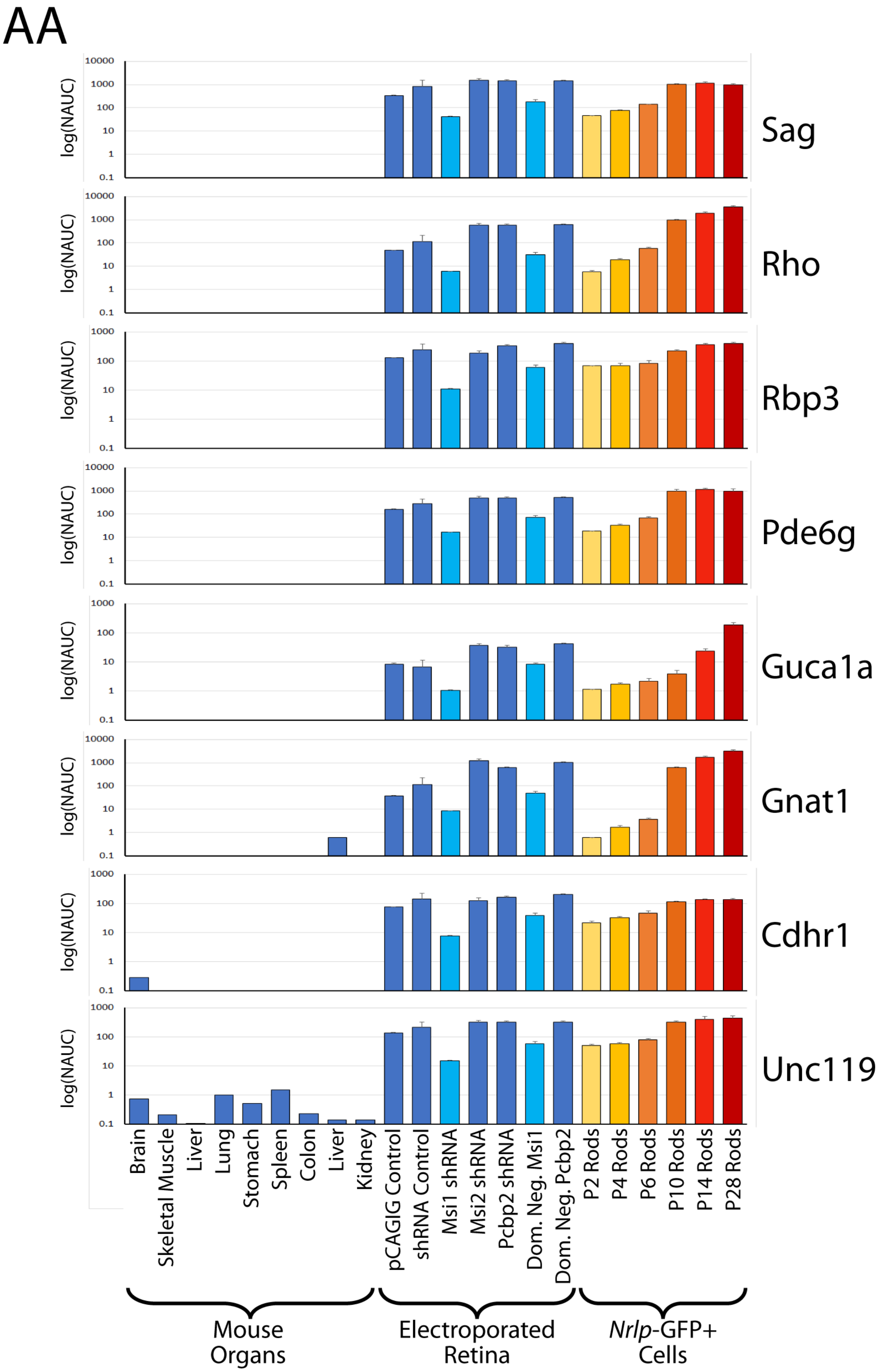

AA
log(NAUC)
Sag
Rho
Rbp3
Pde6g
Guca1a
Gnat1
Cdhr1
Unc119
Brain
Skeletal Muscle
Liver
Lung
Stomach
Spleen
Colon
Liver
Kidney
pCAGIG Control
shRNA Control
Msi1 shRNA
Msi2 shRNA
Pcbp2 shRNA
Dom. Neg. Msi1
Dom. Neg. Pcbp2
P2 Rods
P4 Rods
P6 Rods
P10 Rods
P14 Rods
P28 Rods
Mouse Organs
Electroporated Retina
Nrlp-GFP+ Cells

Supplemental Figure 11. *Msi1* is necessary for rod photoreceptor-specific splicing

(**A**-**Z**) Knockdown of *Msi1* in retina abolishes nearly all rod-specific splicing, while knockdown of *Msi2* and *Pcbp2* have minimal effects on photoreceptor splicing. Expression of a dominant negative *Msi1* protein phenocopies *Msi1* knockdown, although to a lesser degree. Notably, some exons remain unaffected by *Msi1* knockdown, e.g. exons in *Doc2b* (**F**), *Ppp3cc* (**T**), and *Plekhb1* (**U**), while other exons are only partially repressed. (**AA**) Analysis of genes previously associated with photoreceptor development[22] reveals that both *Msi1* knockdown and dominant negative *Msi1* may delay photoreceptor maturation.

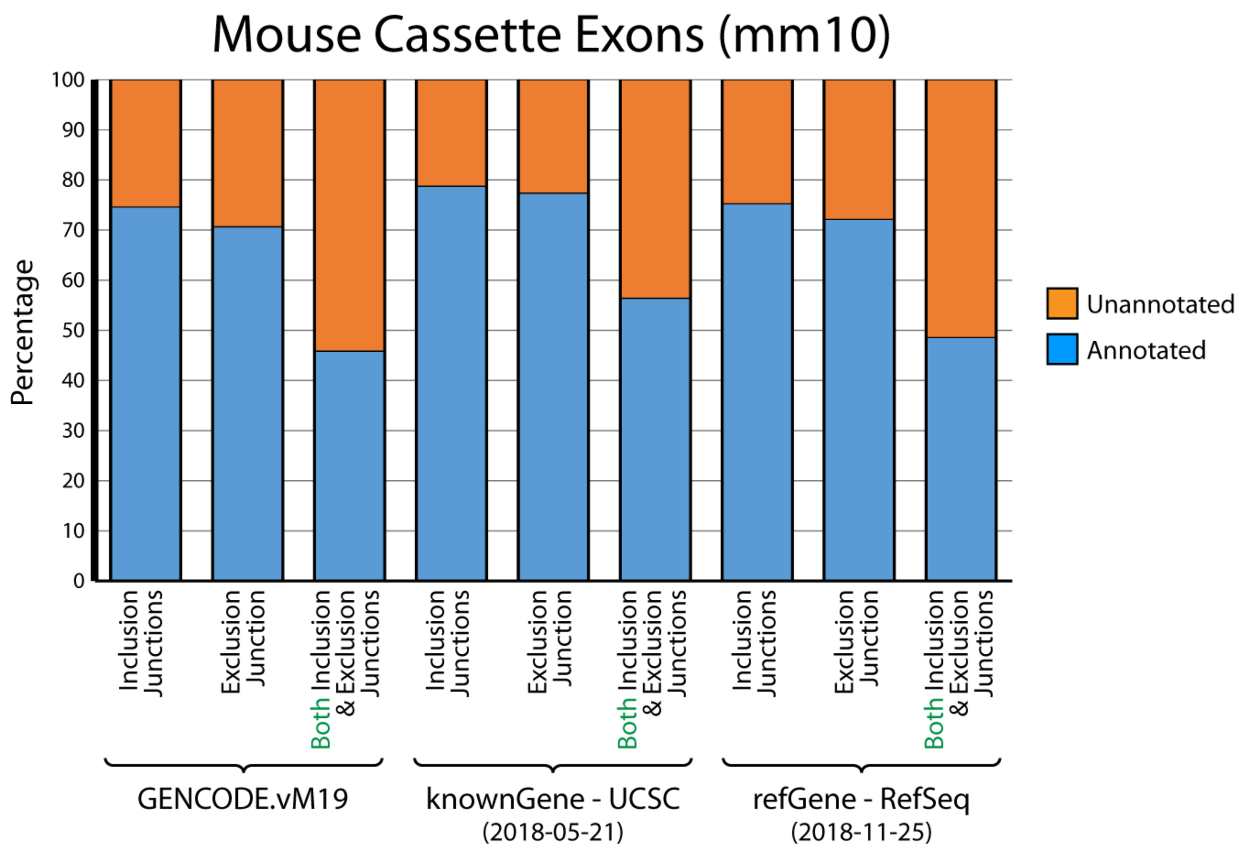
Mouse Cassette Exons (mm10)
Percentage
100
90
80
70
60
50
40
30
20
10
0
Unannotated
Annotated
Inclusion Junctions
Exclusion Junction
Both Inclusion & Exclusion Junctions
Inclusion Junctions
Exclusion Junction
Both Inclusion & Exclusion Junctions
Inclusion Junctions
Exclusion Junction
Both Inclusion & Exclusion Junctions
GENCODE.vM19
knownGene - UCSC
(2018-05-21)
refGene - RefSeq
(2018-11-25)

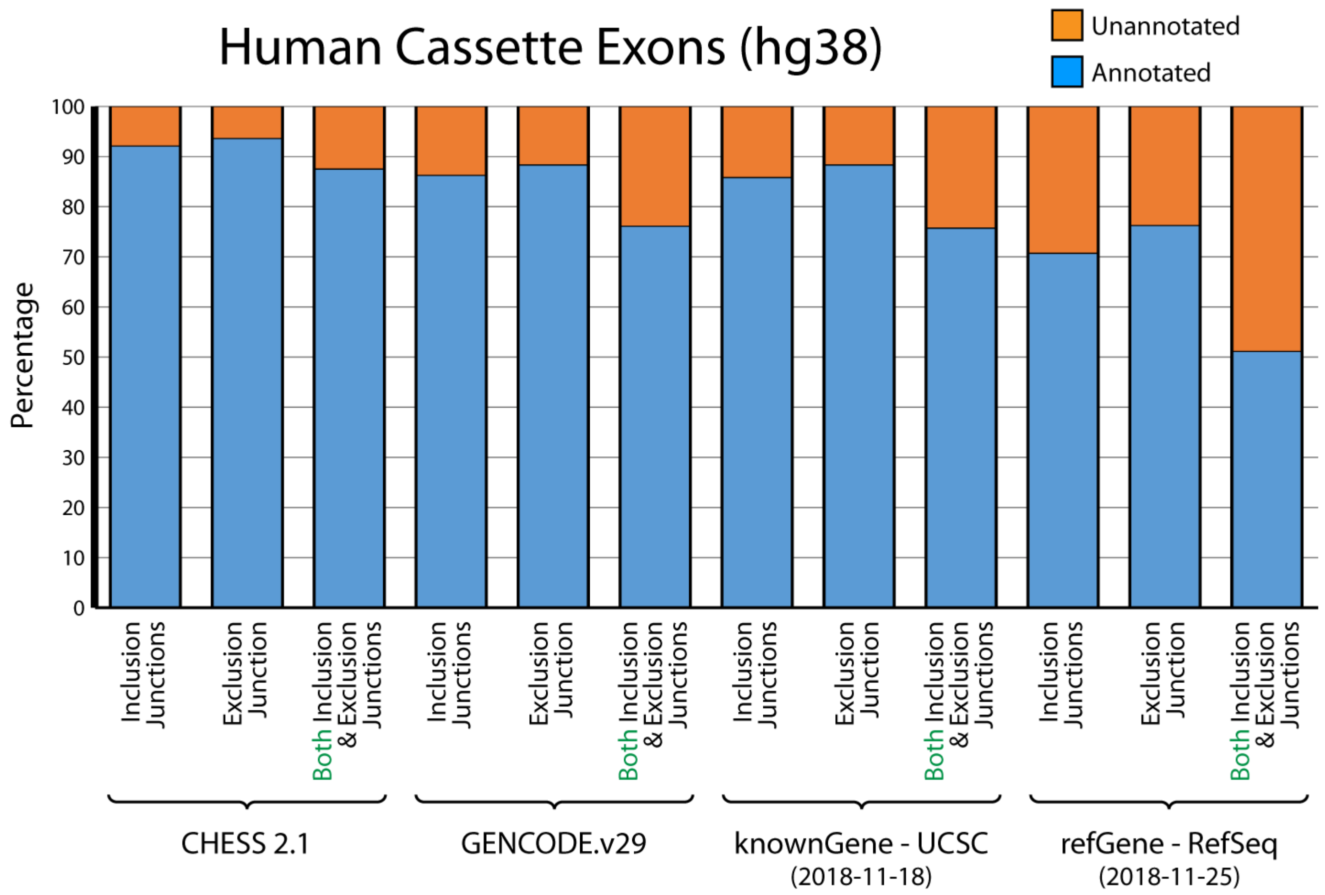
Human Cassette Exons (hg38)
Unannotated
Annotated
Percentage
100
90
80
70
60
50
40
30
20
10
0
Inclusion Junctions
Exclusion Junction
Both Inclusion & Exclusion Junctions
Inclusion Junctions
Exclusion Junction
Both Inclusion & Exclusion Junctions
Inclusion Junctions
Exclusion Junction
Both Inclusion & Exclusion Junctions
Inclusion Junctions
Exclusion Junction
Both Inclusion & Exclusion Junctions
CHESS 2.1
GENCODE.v29
knownGene - UCSC
(2018-11-18)
refGene - RefSeq
(2018-11-25)

Supplemental Figure 12. Many of the exons detected by ASCOT are unannotated

To estimate the percentage of unannotated exons that are identified by ASCOT, we looked at high abundance cassette exons (exons detected in at least 75% of samples in MESA or GTEx and exons where the difference between the maximum PSI and the minimum PSI was >75). For mouse cassette exons, we compared against the GENCODE.vM19, knownGene (2018-05-21), and refGene (2018-11-25) gene reference annotations. For human cassette exons, we compared against the CHESS2.1[23], GENCODE.v29, knownGene (2018-11-18), and refGene (2018-11-25) gene reference annotations. For each gene reference annotation, we determined whether the inclusion junctions or exclusion junction of a given cassette exon was annotated and whether the cassette exon was fully annotated ("Both Inclusion & Exclusion Junctions"). We estimate that ~40-60% of mouse and ~10-30% of human cassette exons identified by ASCOT are unannotated.

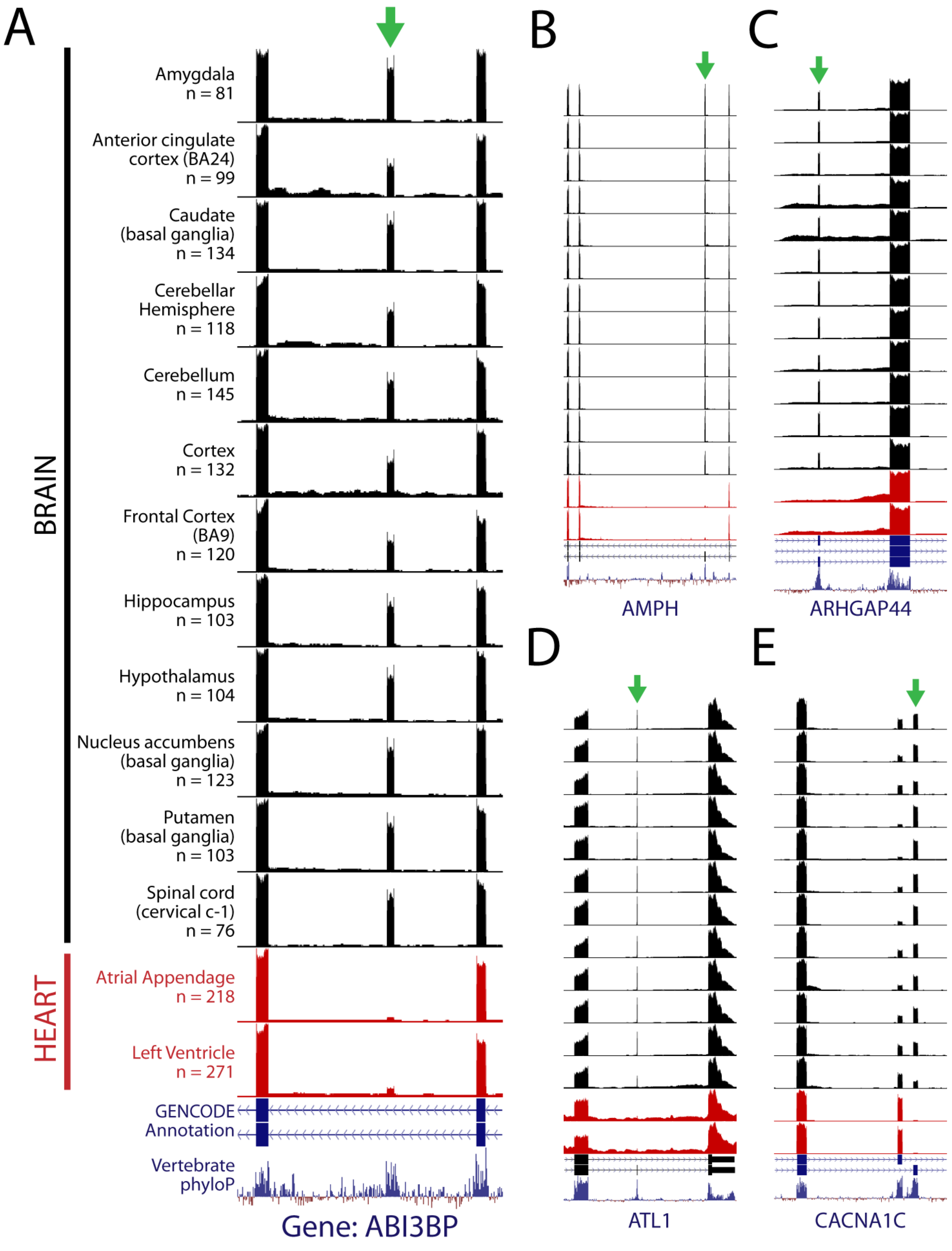
A
B
C
D
E
BRAIN
HEART
Amygdala
n = 81
Anterior cingulate
cortex (BA24)
n = 99
Caudate
(basal ganglia)
n = 134
Cerebellar
Hemisphere
n = 118
Cerebellum
n = 145
Cortex
n = 132
Frontal Cortex
(BA9)
n = 120
Hippocampus
n = 103
Hypothalamus
n = 104
Nucleus accumbens
(basal ganglia)
n = 123
Putamen
(basal ganglia)
n = 103
Spinal cord
(cervical c-1)
n = 76
Atrial Appendage
n = 218
Left Ventricle
n = 271
GENCODE
Annotation
Vertebrate
phyloP
Gene: ABI3BP
AMPH
ARHGAP44
ATL1
CACNA1C

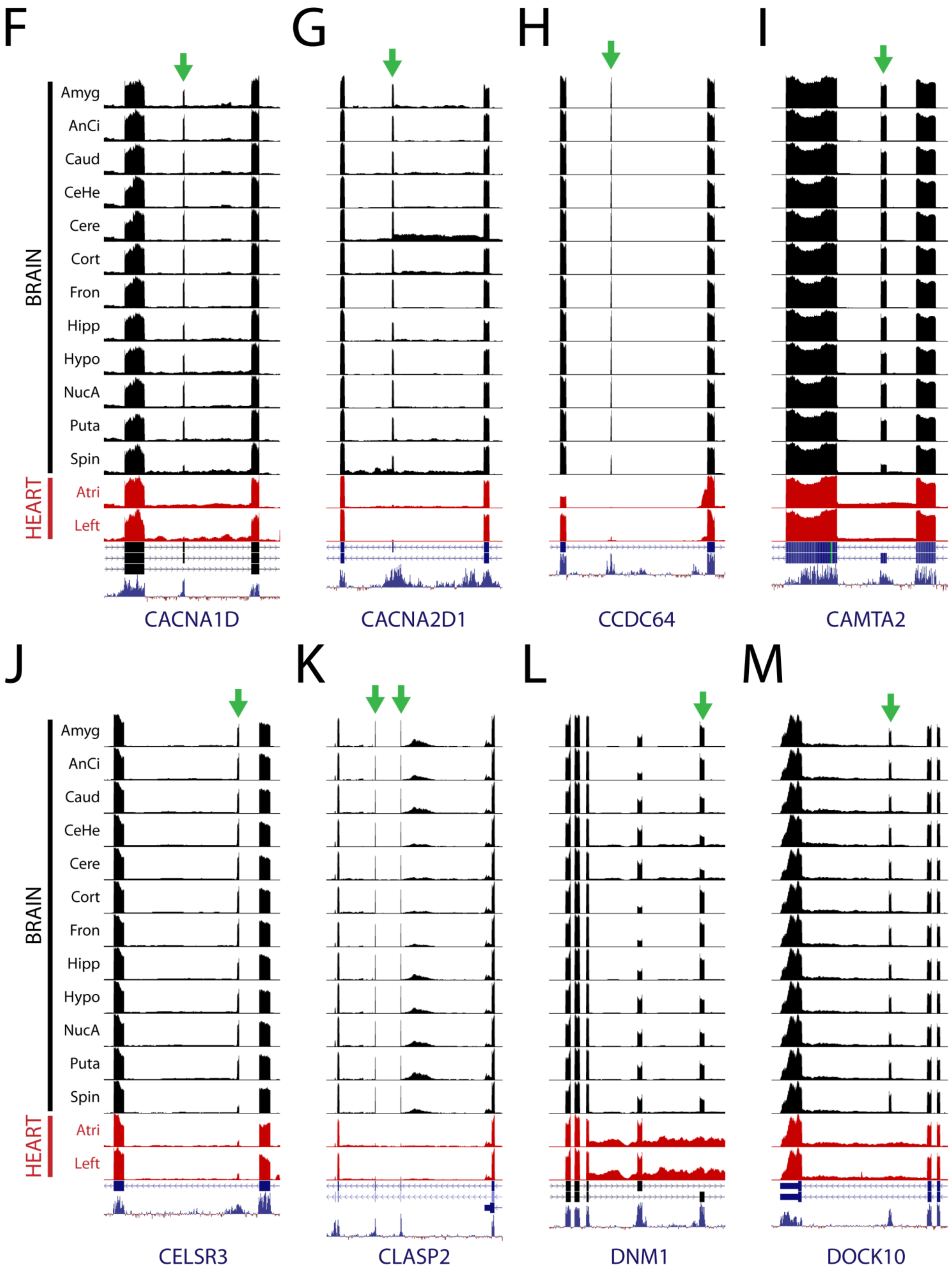

F
G
H
I
J
K
L
M
BRAIN
HEART
Amyg
AnCi
Caud
CeHe
Cere
Cort
Fron
Hipp
Hypo
NucA
Puta
Spin
Atri
Left
CACNA1D
CACNA2D1
CCDC64
CAMTA2
CELSR3
CLASP2
DNM1
DOCK10

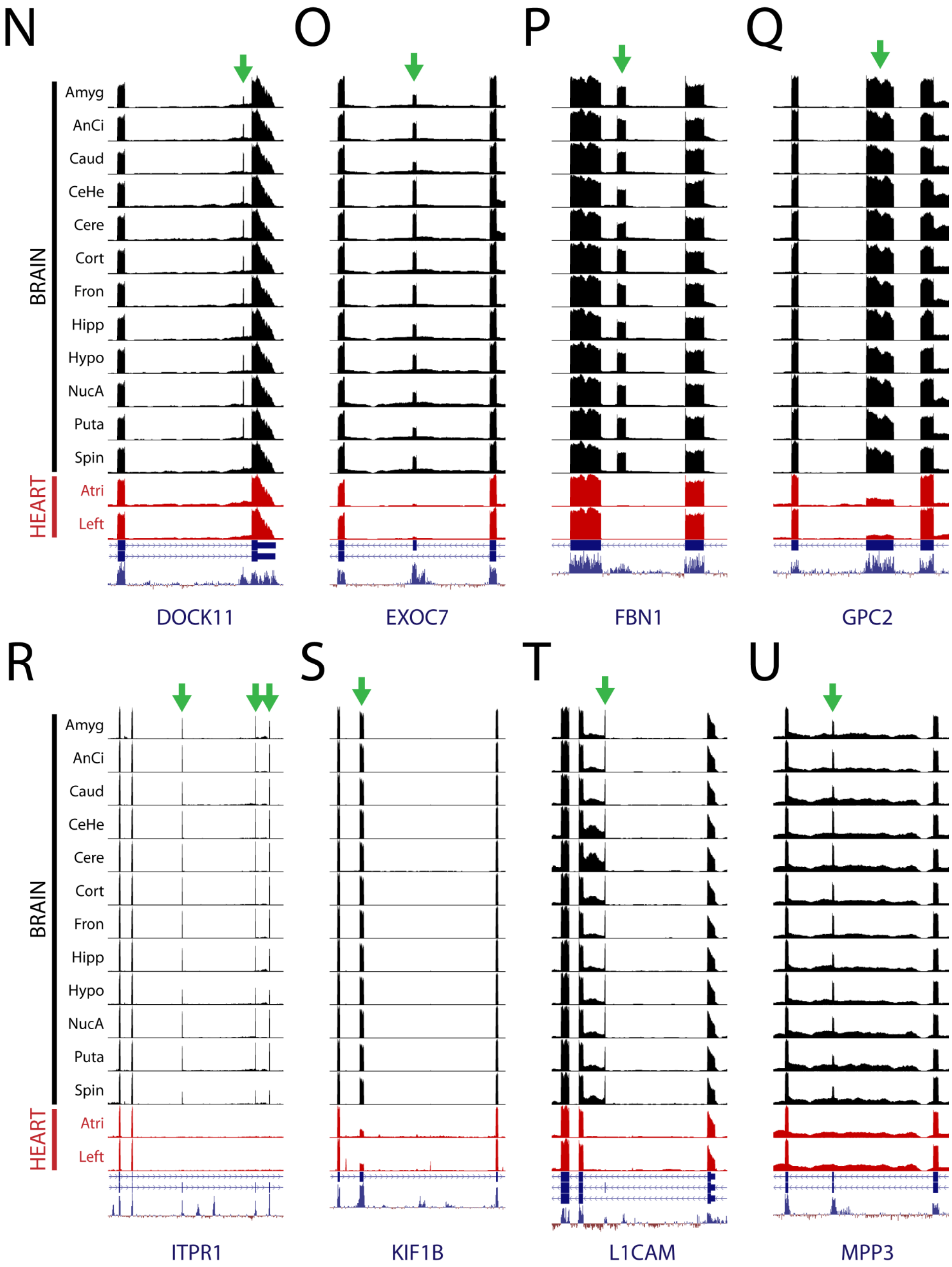
N
O
P
Q
Amyg
AnCi
Caud
CeHe
Cere
Cort
Fron
Hipp
Hypo
NucA
Puta
Spin
Atri
Left
BRAIN
HEART
DOCK11
EXOC7
FBN1
GPC2
R
S
T
U
ITPR1
KIF1B
L1CAM
MPP3

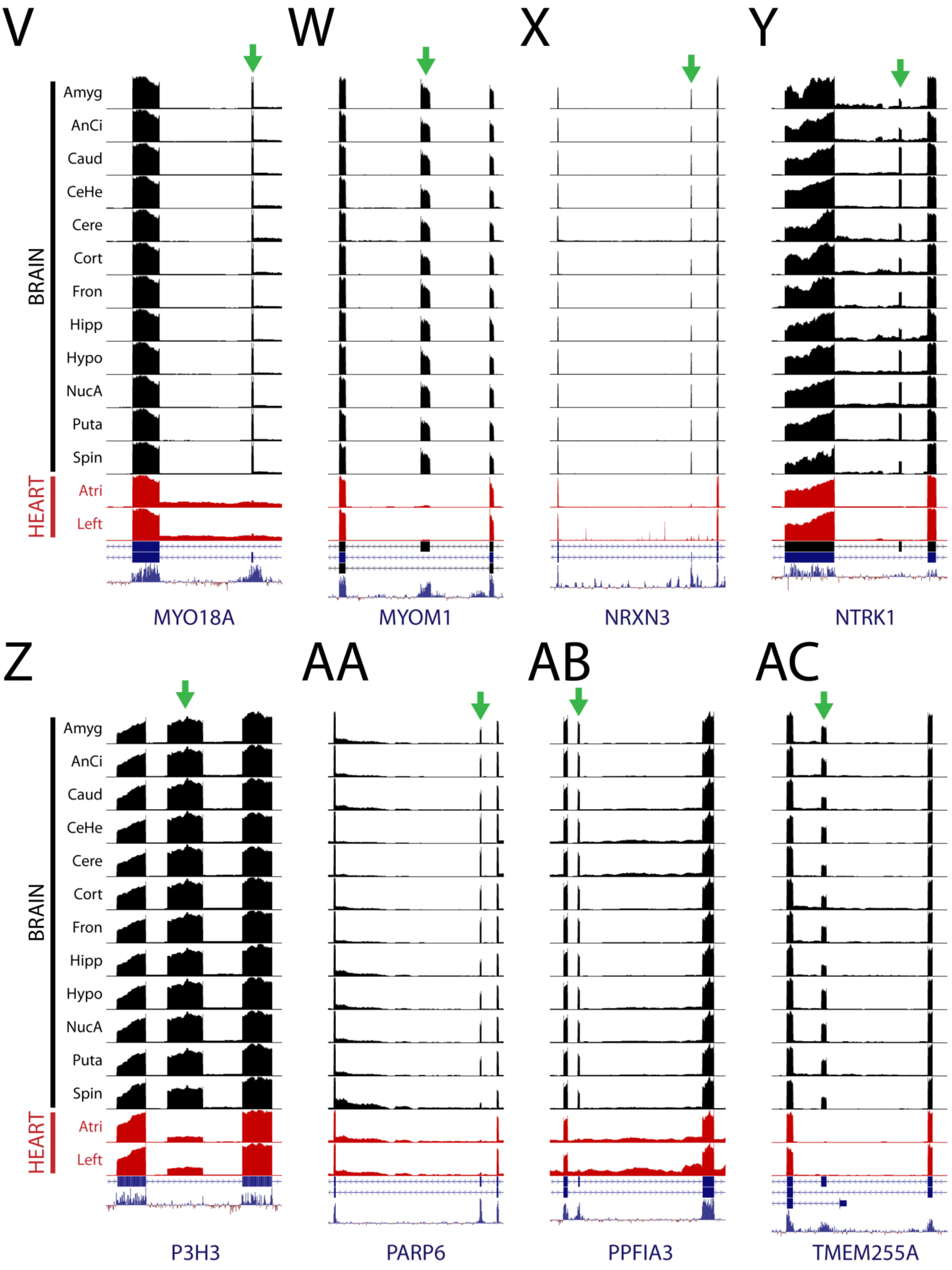

V
W
X
Y
Z
AA
AB
AC
BRAIN
HEART
Amyg
AnCi
Caud
CeHe
Cere
Cort
Fron
Hipp
Hypo
NucA
Puta
Spin
Atri
Left
MYO18A
MYOM1
NRXN3
NTRK1
P3H3
PARP6
PPFIA3
TMEM255A

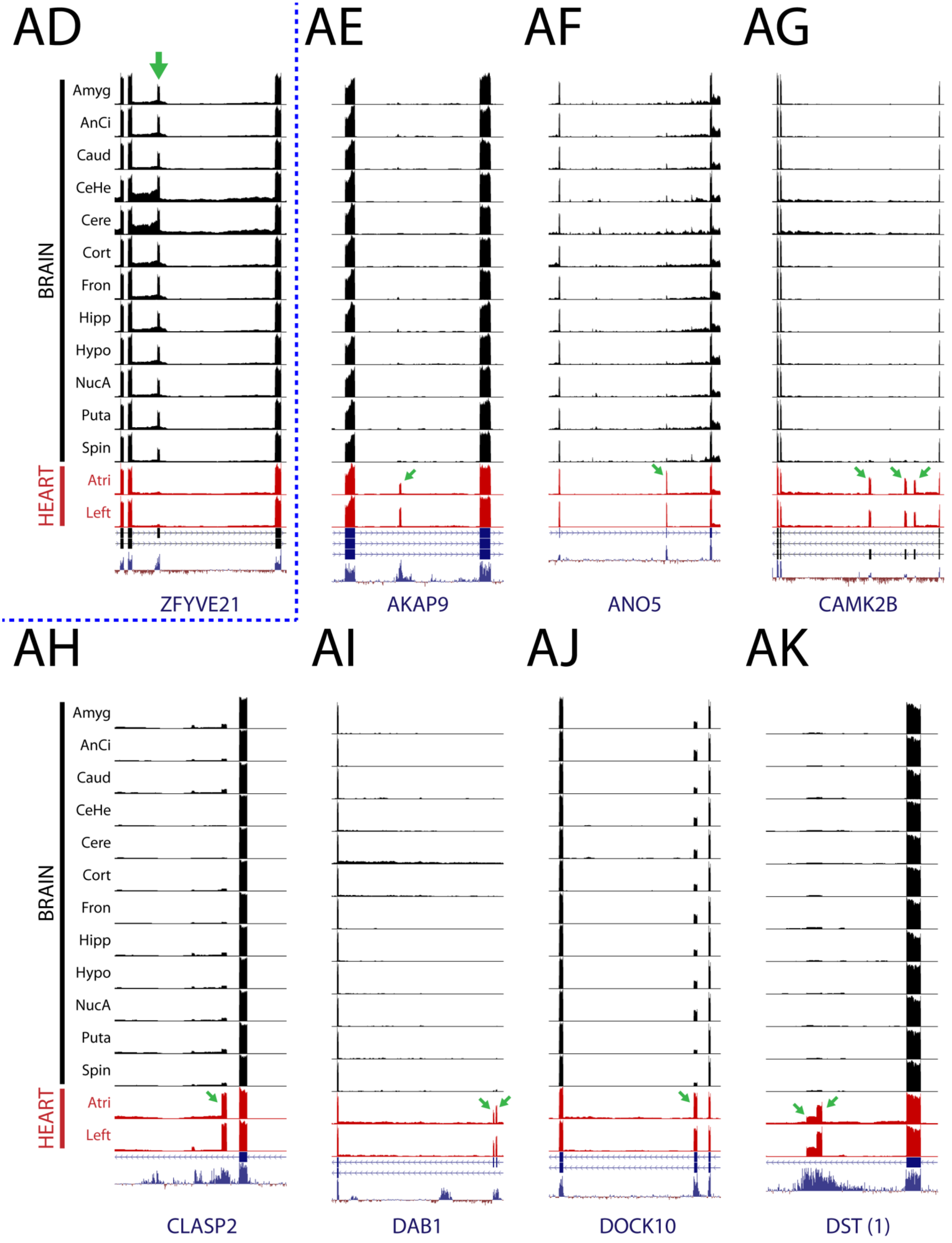
AD
AE
AF
AG
BRAIN
Amyg
AnCi
Caud
CeHe
Cere
Cort
Fron
Hipp
Hypo
NucA
Puta
Spin
HEART
Atri
Left
ZFYVE21
AKAP9
ANO5
CAMK2B
AH
AI
AJ
AK
BRAIN
Amyg
AnCi
Caud
CeHe
Cere
Cort
Fron
Hipp
Hypo
NucA
Puta
Spin
HEART
Atri
Left
CLASP2
DAB1
DOCK10
DST (1)

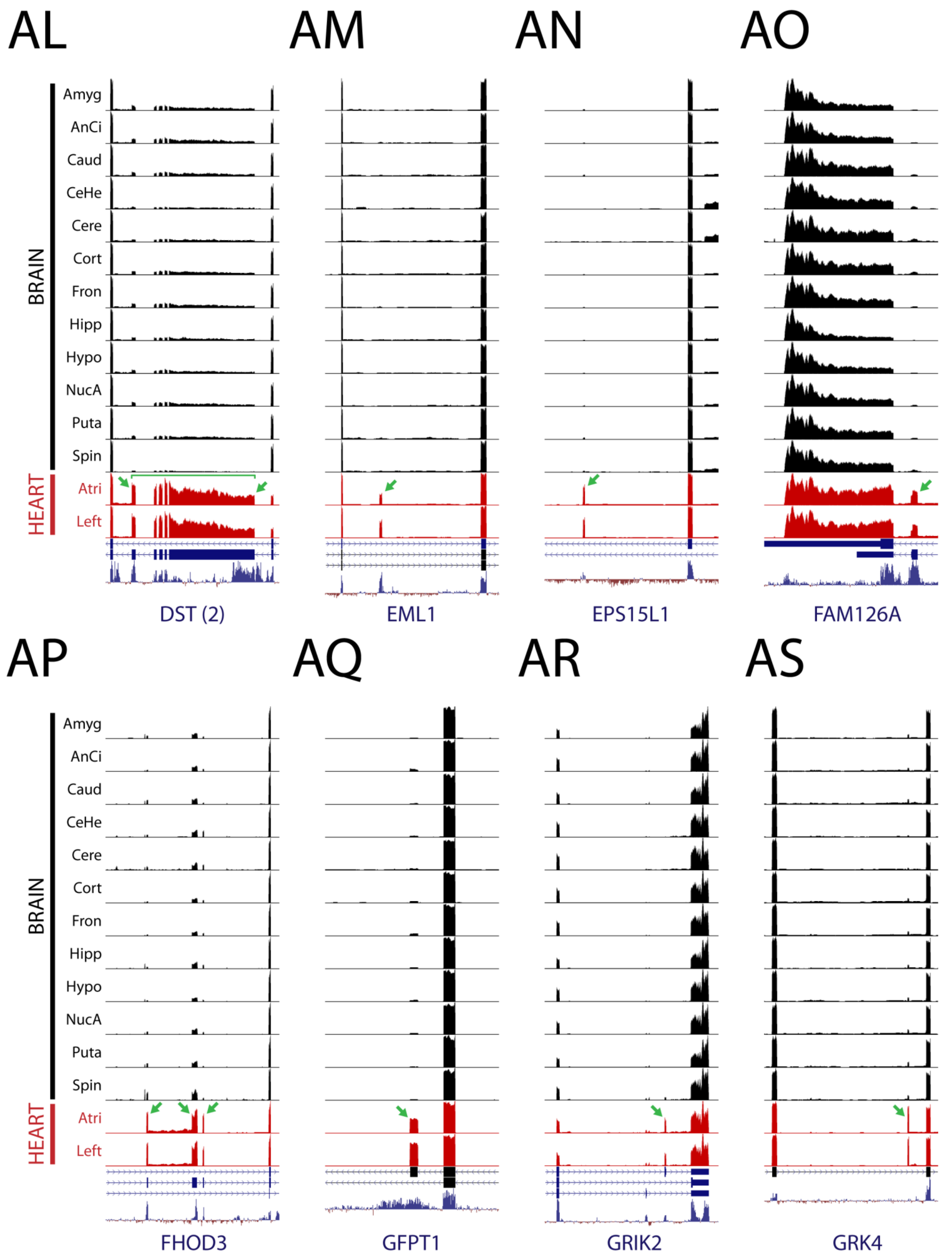
AL
AM
AN
AO
BRAIN
Amyg
AnCi
Caud
CeHe
Cere
Cort
Fron
Hipp
Hypo
NucA
Puta
Spin
HEART
Atri
Left
DST (2)
EML1
EPS15L1
FAM126A
AP
AQ
AR
AS
BRAIN
Amyg
AnCi
Caud
CeHe
Cere
Cort
Fron
Hipp
Hypo
NucA
Puta
Spin
HEART
Atri
Left
FHOD3
GFPT1
GRIK2
GRK4

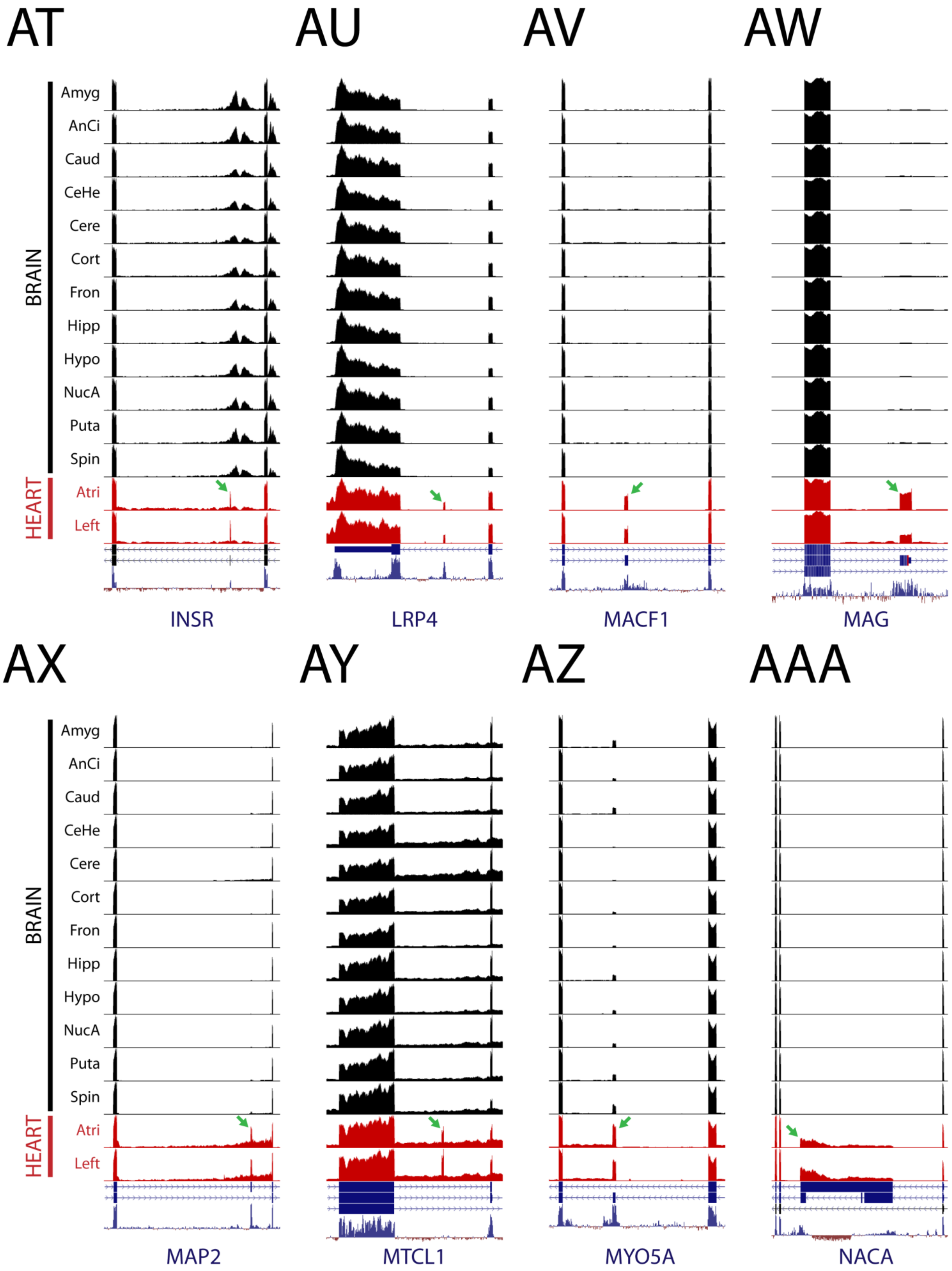
AT
AU
AV
AW
BRAIN
HEART
Amyg
AnCi
Caud
CeHe
Cere
Cort
Fron
Hipp
Hypo
NucA
Puta
Spin
Atri
Left
INSR
LRP4
MACF1
MAG
AX
AY
AZ
AAA
MAP2
MTCL1
MYO5A
NACA

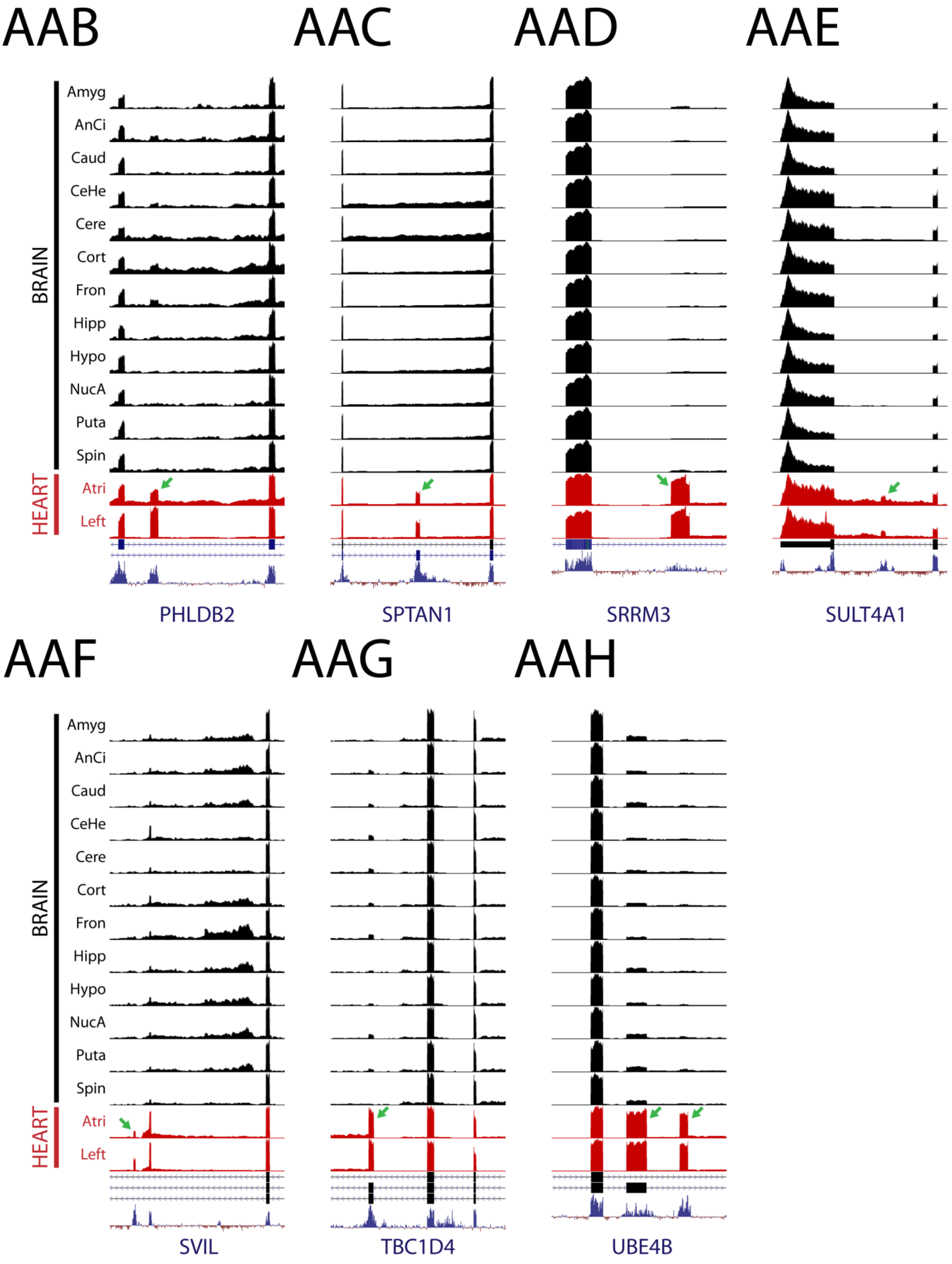
AAB
AAC
AAD
AAE
AAF
AAG
AAH
BRAIN
HEART
Amyg
AnCi
Caud
CeHe
Cere
Cort
Fron
Hipp
Hypo
NucA
Puta
Spin
Atri
Left
PHLDB2
SPTAN1
SRRM3
SULT4A1
SVIL
TBC1D4
UBE4B

Supplemental Figure 13. Dataset clustering can improve the detection of binary splicing events

Alternative splicing algorithms such as MAJIQ and LeafCutter can model complex splicing events that are not analyzed by ASCOT. However, ASCOT can reliably detect simple binary splicing events due to the simplified junction walking search logic. (**A** – **AAH**) 60 examples of alternative exons that are not recorded in the LeafCutter shinyapp (https://leafcutter.shinyapps.io/leafviz/) are shown; 30 exons that are highly specific to brain (**A** – **AD**) and 30 exons that are highly specific to heart (**AE** – **AAH**).

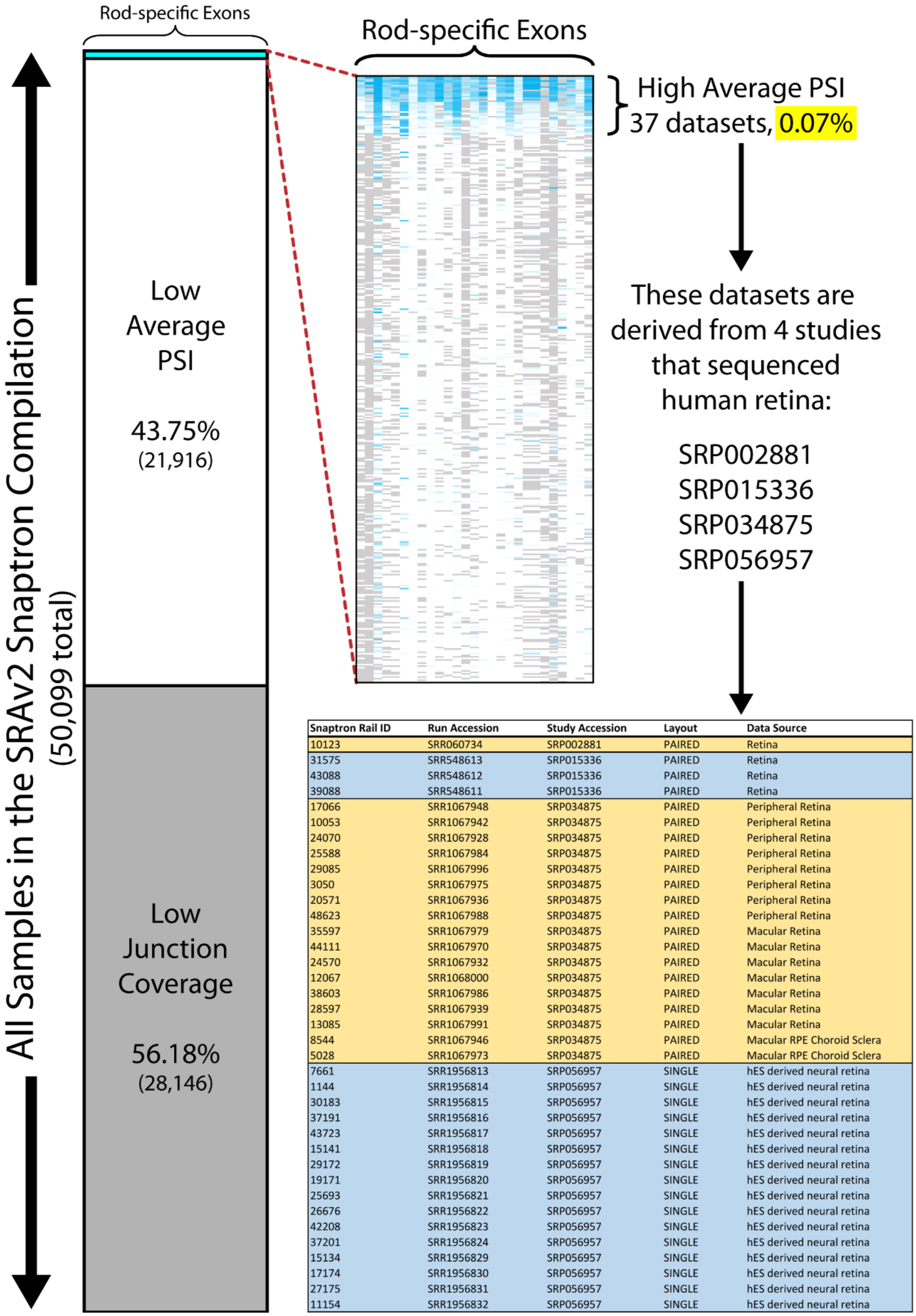

Snaptron Rail ID	Run Accession	Study Accession	Layout	Data Source
10123	SRR060734	SRP002881	PAIRED	Retina
31575	SRR548613	SRP015336	PAIRED	Retina
43088	SRR548612	SRP015336	PAIRED	Retina
39088	SRR548611	SRP015336	PAIRED	Retina
17066	SRR1067948	SRP034875	PAIRED	Peripheral Retina
10053	SRR1067942	SRP034875	PAIRED	Peripheral Retina
24070	SRR1067928	SRP034875	PAIRED	Peripheral Retina
25588	SRR1067984	SRP034875	PAIRED	Peripheral Retina
29085	SRR1067996	SRP034875	PAIRED	Peripheral Retina
3050	SRR1067975	SRP034875	PAIRED	Peripheral Retina
20571	SRR1067936	SRP034875	PAIRED	Peripheral Retina
48623	SRR1067988	SRP034875	PAIRED	Peripheral Retina
35597	SRR1067979	SRP034875	PAIRED	Macular Retina
44111	SRR1067970	SRP034875	PAIRED	Macular Retina
24570	SRR1067932	SRP034875	PAIRED	Macular Retina
12067	SRR1068000	SRP034875	PAIRED	Macular Retina
38603	SRR1067986	SRP034875	PAIRED	Macular Retina
28597	SRR1067939	SRP034875	PAIRED	Macular Retina
13085	SRR1067991	SRP034875	PAIRED	Macular Retina
8544	SRR1067946	SRP034875	PAIRED	Macular RPE Choroid Sclera
5028	SRR1067973	SRP034875	PAIRED	Macular RPE Choroid Sclera
7661	SRR1956813	SRP056957	SINGLE	hES derived neural retina
1144	SRR1956814	SRP056957	SINGLE	hES derived neural retina
30183	SRR1956815	SRP056957	SINGLE	hES derived neural retina
37191	SRR1956816	SRP056957	SINGLE	hES derived neural retina
43723	SRR1956817	SRP056957	SINGLE	hES derived neural retina
15141	SRR1956818	SRP056957	SINGLE	hES derived neural retina
29172	SRR1956819	SRP056957	SINGLE	hES derived neural retina
19171	SRR1956820	SRP056957	SINGLE	hES derived neural retina
25693	SRR1956821	SRP056957	SINGLE	hES derived neural retina
26676	SRR1956822	SRP056957	SINGLE	hES derived neural retina
42208	SRR1956823	SRP056957	SINGLE	hES derived neural retina
37201	SRR1956824	SRP056957	SINGLE	hES derived neural retina
15134	SRR1956829	SRP056957	SINGLE	hES derived neural retina
17174	SRR1956830	SRP056957	SINGLE	hES derived neural retina
27175	SRR1956831	SRP056957	SINGLE	hES derived neural retina
11154	SRR1956832	SRP056957	SINGLE	hES derived neural retina

Supplemental Figure 14. Rod-specific exons in the SRAv2 Snaptron compilation

Screening the SRA for rod-specific exons, we identify 37 datasets (~0.07%) in the SRAv2 Snaptron compilation with rod-specific exons that originated from human retina RNA-Seq.

Supplementary References

1. Wilks, C., Gaddipati, P., Nellore, A. & Langmead, B. Snaptron: querying splicing patterns across tens of thousands of RNA-seq samples. *Bioinformatics* **34**, 114–116 (2018).
2. Vaquero-Garcia, J. *et al.* A new view of transcriptome complexity and regulation through the lens of local splicing variations. *Elife* **5**, e11752 (2016).
3. Terada, Y. *et al.* Novel splice variants of amphiphysin I are expressed in retina. *FEBS Lett.* **519**, 185–90 (2002).
4. Murphy, D., Cieply, B., Carstens, R., Ramamurthy, V. & Stoilov, P. The Musashi 1 Controls the Splicing of Photoreceptor-Specific Exons in the Vertebrate Retina. *PLoS Genet.* **12**, e1006256 (2016).
5. Friedrich, U. *et al.* The Na/K-ATPase is obligatory for membrane anchorage of retinoschisin, the protein involved in the pathogenesis of X-linked juvenile retinoschisis. *Hum. Mol. Genet.* **20**, 1132–42 (2011).
6. Wan, J. *et al.* Dynamic usage of alternative splicing exons during mouse retina development. *Nucleic Acids Res.* **39**, 7920–30 (2011).
7. Ochrietor, J. D. *et al.* Retina-specific expression of 5A11/Basigin-2, a member of the immunoglobulin gene superfamily. *Invest. Ophthalmol. Vis. Sci.* **44**, 4086–96 (2003).
8. Ochrietor, J. D. & Linser, P. J. 5A11/Basigin gene products are necessary for proper maturation and function of the retina. *Dev. Neurosci.* **26**, 380–7 (2004).
9. Clamp, M. F., Ochrietor, J. D., Moroz, T. P. & Linser, P. J. Developmental analyses of 5A11/Basigin, 5A11/Basigin-2 and their putative binding partner MCT1 in the mouse eye. *Exp. Eye Res.* **78**, 777–89 (2004).
10. Riazuddin, S. A. *et al.* A splice-site mutation in a retina-specific exon of BBS8 causes nonsyndromic retinitis pigmentosa. *Am. J. Hum. Genet.* **86**, 805–12 (2010).
11. Murphy, D., Singh, R., Kolandaivelu, S., Ramamurthy, V. & Stoilov, P. Alternative Splicing Shapes the Phenotype of a Mutation in BBS8 To Cause Nonsyndromic Retinitis Pigmentosa. *Mol. Cell. Biol.* **35**, 1860–70 (2015).
12. Bowne, S. J. *et al.* Why do mutations in the ubiquitously expressed housekeeping gene IMPDH1 cause retina-specific photoreceptor degeneration? *Invest. Ophthalmol. Vis. Sci.* **47**, 3754–65 (2006).
13. Tasic, B. *et al.* Adult mouse cortical cell taxonomy revealed by single cell transcriptomics. *Nat. Neurosci.* **19**, 335–46 (2016).

14. Clark, B. *et al.* Comprehensive analysis of retinal development at single cell resolution identifies NFI factors as essential for mitotic exit and specification of late-born cells. *bioRxiv* (2018). doi:10.1101/378950
15. Ling, J. P. *et al.* PTBP1 and PTBP2 Repress Nonconserved Cryptic Exons. *Cell Rep.* **17**, 104–113 (2016).
16. McClory, S. P., Lynch, K. W. & Ling, J. P. HnRNP L represses cryptic exons. *RNA* **24**, 761–768 (2018).
17. Ohyama, T. *et al.* Structure of Musashi1 in a complex with target RNA: the role of aromatic stacking interactions. *Nucleic Acids Res.* **40**, 3218–31 (2012).
18. Zearfoss, N. R. *et al.* A conserved three-nucleotide core motif defines Musashi RNA binding specificity. *J. Biol. Chem.* **289**, 35530–41 (2014).
19. Iwaoka, R. *et al.* Structural Insight into the Recognition of r(UAG) by Musashi-1 RBD2, and Construction of a Model of Musashi-1 RBD1-2 Bound to the Minimum Target RNA. *Molecules* **22**, (2017).
20. Sloan, C. A. *et al.* ENCODE data at the ENCODE portal. *Nucleic Acids Res.* **44**, D726-32 (2016).
21. Li, N. *et al.* The Msi Family of RNA-Binding Proteins Function Redundantly as Intestinal Oncoproteins. *Cell Rep.* **13**, 2440–2455 (2015).
22. Blackshaw, S. *et al.* Genomic analysis of mouse retinal development. *PLoS Biol.* **2**, E247 (2004).
23. Pertea, M. *et al.* CHESS: a new human gene catalog curated from thousands of large-scale RNA sequencing experiments reveals extensive transcriptional noise. *Genome Biol.* **19**, 208 (2018).
